# Comparative proteomic analysis of metabolically labelled proteins from *Plasmodium falciparum *isolates with different adhesion properties

**DOI:** 10.1186/1475-2875-5-67

**Published:** 2006-08-03

**Authors:** Yang Wu, Alister Craig

**Affiliations:** 1 Liverpool School of Tropical Medicine, Pembroke Place, Liverpool L3 5QA, UK

## Abstract

The virulence of *Plasmodium falciparum *relates in part to the cytoadhesion characteristics of parasitized erythrocytes but the molecular basis of the different qualitative and quantitative binding phenotypes is incompletely understood. This paucity of information is due partly to the difficulty in working with membrane proteins, the variant nature of these surface antigens and their relatively low abundance. To address this two-dimensional (2D) protein profiles of closely related, but phenotypically different laboratory strains of *P. falciparum *have been characterized using proteomic approaches. Since the mature erythrocyte has no nucleus and no protein synthesis capability, metabolic labelling of proteins was used to selectively identify parasite proteins and increase detection sensitivity.

A small number of changes (less than 10) were observed between four different *P. falciparum *laboratory strains with distinctive cytoadherence properties using metabolic labelling, with more parasite protein changes found in trophozoite iRBCs than ring stage. The combination of metabolic labelling and autoradiography can therefore be used to identify parasite protein differences, including quantitative ones, and in some cases to obtain protein identifications by mass spectrometry. The results support the suggestion that the membrane protein profile may be related to cytoadherent properties of the iRBCs. Most changes between parasite variants were differences in iso-electric point indicating differential protein modification rather than the presence or absence of a specific peptide.

## Background

The virulence of the human malaria parasite *Plasmodium falciparum *is believed to relate in part to adhesion of parasitized erythrocytes to postcapillary venular endothelium and other uninfected blood cells. At the molecular level, surface antigens from infected erythrocytes interact with several host receptors such as CD36 [1-3], Intercellular adhesion molecule-1 (ICAM-1) (CD54) [4], thrombospondin [5], E-selectin (CD62E), vascular cell adhesion molecule VCAM-1 (CD106) [6], P-selectin (CD62P) [7], α_v_β_3_-integrin [8], platelet endothelial cell adhesion molecule-1 [PECAM-1 (CD31)] [9], Chondroitin sulfate A (CSA) [10], Complement receptor-1 (CR-1)),(CD35) [11], Heparan sulphate (HS)-like Glycosaminoglycan (GAG) [12], ABO blood group antigens [13] resulting in the rosetting, agglutination [14] and cytoadherence of iRBC. A parasite-derived adhesion molecule that mediates these interactions has been identified as *P. falciparum *erythrocyte membrane protein 1 (PfEMP1), which is encoded by the *var *multigene family [15-17]. There are about 60 *var *genes per parasite genome [18] that switch on expression in a mutually exclusive manner and contain copies of motifs that bind to diverse host receptors. Direct association of specific adhesive phenotype with disease has not been easy to demonstrate, except for pregnancy-associated malaria, in which the ability of iRBC to bind to placental receptors (such as CSA) is closely related to poor maternal and neonatal outcome [19]. A number of other parasite proteins associated with the erythrocyte membrane and exported on the surface of parasitized erythrocytes have been reported, including, Rifin, Stevor, ring surface protein (RSP) 1/2, (for a review see [20]) and, more recently, Surfins [21]. These proteins form part of a group of *P. falciparum *proteins that contain signals for export from the parasitophorous vacuole [22,23]. Further bioinformatic analysis has proposed over 200 proteins that contain these signals and this behaviour appears to have been adopted much more extensively in *P. falciparum *than in other *Plasmodium *species [24], perhaps in part due to its requirement to undergo antigenic variation and cytoadherence.

There is a correlation between parasite clones of different antigenic type and cytoadherence [25,26], rosette formation and pathogenesis, which has led to the studies on the diversity of these properties in *P. falciparum *isolates by testing binding to purified host proteins or cell lines. The parental *P. falciparum *line IT4/25/5 was originally selected by binding to ICAM-1, C32 amelanotic melanoma cells and Human umbilical vein endothelial cells (HUVEC), followed by sub-cloning to produce ItG [27], A4 and C24 [25]. These parasite clones have distinctive cytoadherence properties [28,29]. ItG is a high avidity ICAM-1 and CD36 binder, A4 has high avidity for CD36 and lower avidity to ICAM-1, and C24 binds strongly to CD36 with high avidity but not to ICAM-1. An unrelated parasite line, 3D7, is a low binding line, which has low avidity for CD36 and does not bind ICAM-1. However, the molecular basis underpinning these distinctive binding phenotypes and the molecules involved, other than PfEMP1, are poorly understood. Therefore, the very high resolving power of 2D autoradiography of metabolically-labelled proteins has been used to investigate these genotypically related clones that exhibit distinct adhesion phenotypes to identify the repertoire of molecules involved.

*P. falciparum *infection is a sophisticated system including adaptation and interaction of the human erythrocyte and the parasite. At the early ring stage, very few parasite proteins have been synthesized and so the contribution of the ring-stage parasite to the overall protein biomass of the infected erythrocyte is much less than the human proteins, making it hard to distinguish differences between parasite strain protein profiles at this stage. However, the mature human erythrocyte has no nucleus, no protein synthesis capability and no protein trafficking machinery. Metabolic labelling of proteins of *P. falciparum *can easily indicate parasite protein changes. Moreover, metabolic labelling is a more sensitive method for protein detection; the detection limit for silver stained protein spot is 200 ng while the limit for autoradiography of radioisotope labelled protein is 1 ng. In this study, metabolic incorporation was used to label cultured isolates of *P. falciparum *with [^35^S]methionine combined with biochemical enrichment of the infected erythrocyte membrane compartment. The objectives of this study were to use proteomic approaches to obtain a snapshot of *P. falciparum *infection at the protein level in the iRBC membrane and free parasite fractions, compare specific parasite strains to find molecules differentially expressed in parasite isolates with defined adhesion properties and to identify the proteins of interest using MALDI-TOF.

## Materials and methods

### Parasite culture

The *P. falciparum *isolates used in this study were: ItG [27] A4, C24 [25] and 3D7 [30,31]. Parasites were cultured *in vitro *in group O^+ ^human erythrocytes using previously described conditions [32]. Briefly, parasites were cultured in RPMI1640 medium (supplemented with 37.5 mM HEPES, 7 mM D-glucose, 6 mM NaOH, 25 μg ml^-1 ^gentamicin sulphate, 2 mM L-glutamine and 10% human serum) at a pH of 7.2 in a gas mixture of 96% nitrogen, 3% carbon dioxide and 1% oxygen. To minimize the effect of antigenic switching in culture, a batch of stabilates was prepared from post-selection cultures and used for no more than three weeks. To obtain specific life cycle stages of *P. falciparum*, 5% sorbitol treatment or Plasmagel flottation was used. For this study, the trophozoites were obtained 20–24 hours after invasion and ring stages were obtained 6–10 hours after invasion.

### Two-dimensional electrophoresis

Synchronized parasite culture was incubated in 0.05% saponin for 10 minutes (min) in PBS on ice to lyse erythrocytes. The lysate was collected by centrifugation at 10000 × g for 5 min and washed three times with 10 mM Tris-HCl pH7.4 with 1× protease inhibitor Cocktail (Roche) until RBC ghosts were colourless. The lysate was divided into two fractions; red cell ghosts were collected from surface lysate and free parasite pellet from the bottom of the lysate. The two fractions were washed twice more with Tris-HCl buffer, pelleted and solubilized in 2D rehydration buffer [8 M urea, 2 M thiourea, 2% CHAPS, 65 mM dithiothreitol (DTT), and 0.5% ampholyte pH4-7 or 3–10]. For RBC ghost fraction, 50 μl chaotropic membrane extraction reagent 2 (Sigma) 5 M urea, 2 M thiourea, 40 mM Tris base, 2% CHAPS, 2% SB3-10 and 25 μl/ml of tributylphosphine (TBP) were added to help solubilize the erythrocyte membranes, and then solubilized in 2D rehydration buffer. Then the sample was vortexed, sonicated on ice 10 times for five seconds followed by centrifugation at 15000 × g for 10 min and the supernatant was subjected to 2D electrophoresis. The iso-electric focusing (IEF) was performed with pre-cast Amersham immobiline Drystrip gels using IPGphor IEF Unit (Amersham) initially for 10 h for 30 v, followed by 40 min at 200 v, 1 h at 500 v, 4 h at 2000 v and terminated with 8 h at 8000 v; a total of 80,000 vh. The focused strips were equilibrated in 10 ml equilibration solution (50 mM Tris-HCl, pH6.8, 6 M urea, 30% glycerol, 2% Sodium dodecyl sulphate (SDS)) with reducing agent of 1% DTT for 10 min, followed by 10 ml equilibration solution with 4.5% iodoacetamide for another 10 min. The strips were then washed twice briefly with 1 × SDS gel running buffer and loaded on 10% or 12.5% SDS-polyacrylamide gel electrophoresis (PAGE) gels for second dimension separation. The gels were usually run overnight using a Laemmli buffer system [33]. Gels were stained either with Coomassie brilliant blue or silver nitrate according to a previously published protocol [34].

### Metabolic labelling and fluorography

Parasites grown to specific developmental stages were adjusted to 10% parasitaemia and cells were washed with serum-free RPMI 1640 medium without methionine three times. *In vitro *metabolic labelling was carried out in methionine-free RPMI medium by the addition of 50 μCi/ml [^35^S] methionine for four hours under standard culture conditions. The reaction was stopped by removing the radioisotope by centrifugation and washing. To extract proteins from the radio-labelled culture, saponin was added to a final concentration of 0.15% at 4°C for 10 min to lyse the erythrocytes. Following centrifugation at 15,000 g at 4°C for 10 min., the iRBC ghost and free-parasite pellet were collected separately, washed three times using RPMI 1640 without serum and two times of 10 mM Tris-HCl pH7.4 until colourless. The samples were pelleted and subjected to 2D-electrophoresis. After electrophoresis, the gel was fixed and stained with Coomassie Blue, immersed in Amplify (Amersham) for 30 min and dried for fluorography. Following staining with Coomassie Blue or silver nitrate, 2D gel images were acquired using a Powerscan scanner and differential analysis was performed using 2D Evolution software (Nonlinear, Newcastle, UK).

### Spot picking and in gel digestion

SDS-PAGE gels were Coomassie Brilliant Blue-stained and protein spots of interest either from the membrane enriched fractions (Figure 7) or free parasite fraction (Figure 8) were excised and put into Eppendorf Ultra Pure 1.5 ml centrifuge tube (to avoid contamination and false results from lower-grade plastics). The bands were then cut into 1 mm^3 ^cubes and rinsed twice in 200 μl MilliQ water for 15 minutes. The gel slices were dehydrated by the addition of 100 μl of 50% (v/v) acetonitrile/water and incubated at room temperature for 10 minutes, and the dehydrants removed. 100 μl of ammonium bicarbonate (50 mM) was then added to each sample and incubated again at room temperature for 10 minutes. These last two steps were repeated. After removal of the ammonium bicarbonate the gel fragments were incubated in 10 μl of sequence grade trypsin (Promega) (10 μg/ml in 50 mM ammonium bicarbonate) for 18 hours at 37°C and then centrifuged. The supernatant was retained and the gel pellet treated with 20 μl of 70% acetonitrile (v/v in water) for 10 minutes at room temperature. The supernatant from this step was then removed and pooled with the previous supernatant. The combined supernatant was dried in a rotational vacuum concentrator (RVC2-18), resuspended in 15 μl water, dried again and resuspended in 12 μl of 10% acetonitrile and 0.1% formic acid (in water).

### MALDI-TOF mass spectrometry

Samples were loaded in a sandwich manner by adding and immediately removing 0.5 μl matrix [10 mg cyano-4-hydroxycin-namic acid (Sigma) in 1 ml 50% acetonitrile and 0.05% trifluoroacetic acid (TFA)] onto a stainless steel target. Then 0.5 μl of prepared sample was spotted directly onto the target and an equal volume of matrix was added. High-resolution spectra were obtained using a Axima-CFR plus MALDI ToF instrument (Kratos Analytical, Manchester, UK) in reflectron mode. External calibration was performed using a mixed 3 point standards (Angiotensin II, 1046.54, ACTH fragment 18–39, 2465.19, Bradykinin gragment 1–7, 757.39, Sigma) adjacent to the samples. Acquisition and data processing are controlled by Launchpad software (Kratos Analytical, Manchester, UK).

### Database searching

Protein identification was performed by sending trypsin digested peptide masses to three databases (NCBI, MSDA and Swissprot) separately using the MASCOT Peptide Mass Fingerprinting program [35]. All the modifications listed in the searching programme were considered individually as a parameter for the search. The monoisotopic masses were used and the mass tolerance was set to 0.5Da.

## Results and discussion

**Figure 1:**
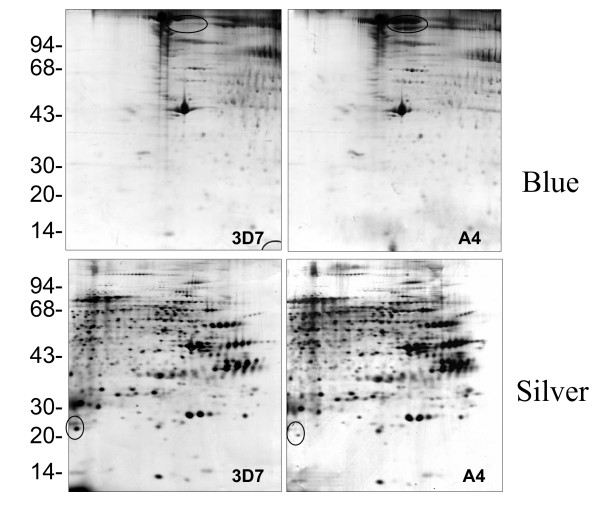
2D electrophoresis profile of Coomassie Blue (upper) and silver (lower) stained iRBC ghosts from 3D7 (left) and A4 (right). The first dimension was run on pH4-7 IEF strips followed by 12.5% SDS-PAGE. The two major changes in the protein profiles are ringed (see text for details). High quality tif files showing the original 2D gels are available for 3D7 iRBC ghosts stained with Coomassie Blue (Additional file 1) and Silver (Additional file 2), and for A4 iRBC ghosts stained with Coomassie Blue (Additional file 3) and silver (Additional file 4).

**Figure 2:**
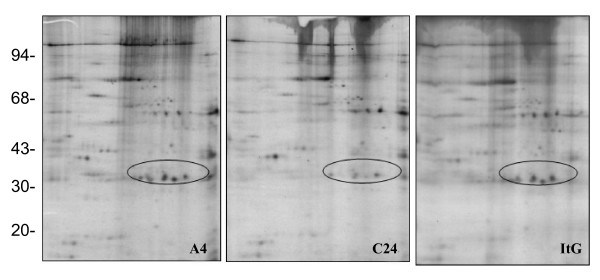
Autoradiograph of proteins from ring stage iRBC ghosts from A4, C24 and ItG. At 6 hours after invasion, parasites were metabolic labelled by 50 μCi/ml [^35^S] methionine for 4 hours. IRBC ghosts were separated from the free parasite fraction and run on pH 4-7 IEF strips followed by 10% SDS-PAGE. Gels were stained with Coomassie Blue, dried and exposed to x-ray film. Marked circles show proteins with at least three fold changes in expression compared with C24. High quality tif files showing the original 2D gels are available for A4 (Additional file 5), C24 (Additional file 6) and ItG (Additional file 7) iRBC ghosts from ring stages.

**Figure 3:**
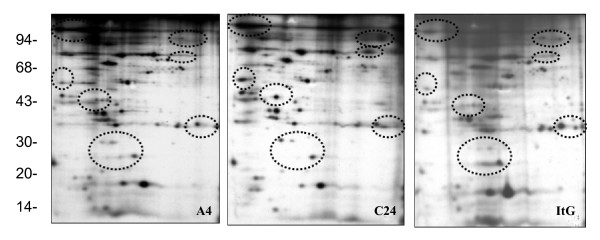
Autoradiograph of proteins from trophozoite stage iRBC ghosts from A4, C24 and ItG. At 20 hours after invasion, parasites were metabolic labelled with 50 μCi/ml [^35^S] methionine for 4 hours. IRBC ghosts were separated from the free parasite fraction and run on pH 4-7 IEF strips followed by 12.5% SDS-PAGE. Gels were stained with Coomassie Blue, dried and exposed to x-ray film. Marked circles show proteins with at least three fold changes in expression compared with C24. High quality tif files showing the original 2D gels are available for A4 (Additional file 8), C24 (Additional file 9) and ItG (Additional file 10) iRBC ghosts from trophozoite stages.

**Figure 4:**
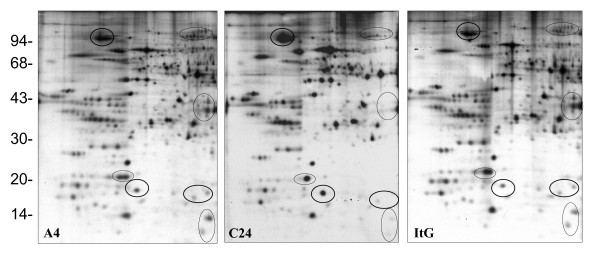
Autoradiograph of proteins from ring stage, free parasite fractions of A4, C24 and ItG. 10 hours after invasion and metabolic labelling, free parasites were separated from IRBC ghosts and run on pH 4-7 IEF strips followed by 12.5% SDS-PAGE. Marked circles show proteins with at least three fold changes in expression compared with C24. High quality tif files showing the original 2D gels are available for A4 (Additional file 11), C24 (Additional file 12) and ItG (Additional file 13) free parasites from ring stages.

**Figure 5:**
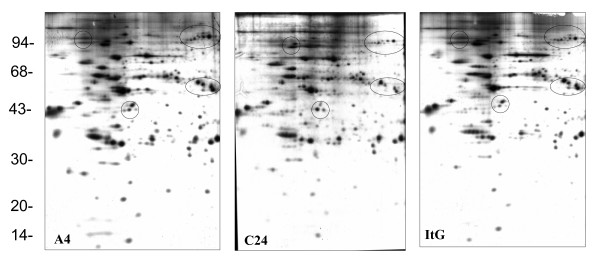
Autoradiograph of proteins from trophozoite stage free parasite fractions of A4, C24 and ItG. 24 hours after invasion and metabolic labelling, free parasites were separated from IRBC ghosts and run on pH 4-7 IEF strips followed by 12.5% SDS-PAGE. Marked circles show proteins with at least three fold changes in expression compared with C24. High quality tif files showing the original 2D gels are available for A4 (Additional file 14), C24 (Additional file 15) and ItG (Additional file 16) free parasites from trophozoite stages.

**Figure 6:**
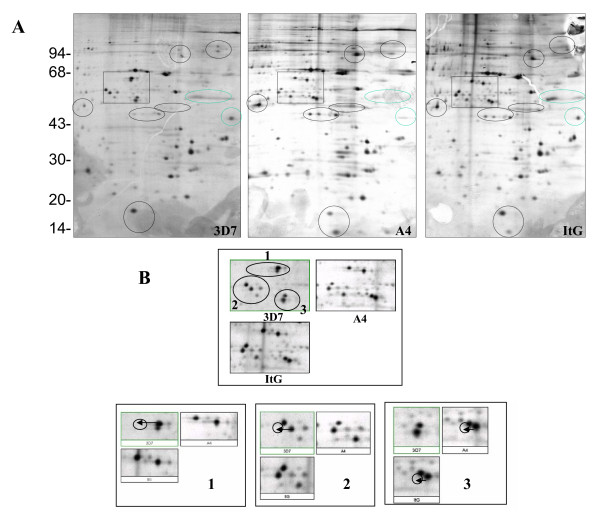
(A). Autoradiograph of proteins from trophozoite stage iRBC infected with 3D7, A4 and ItG. At 20 hours after invasion, parasites were metabolic labelled with 50 μCi/ml [^35^S] methionine for 4 hours. iRBC ghosts were separated from the free parasite fraction and run on pH 4-7 IEF strips followed by 12% SDS-PAGE. Gels were stained with Coomassie Blue, dried and exposed to x-ray film. Marked circles show proteins with at least three fold changes in expression compared with 3D7. High quality tif files showing the original 2D gels of metabolic labelled iRBC ghosts from parasite lines 3D7 (Additional file 17), A4 (Additional file 18) and ItG (Additional file 19) available. (B). Enlarged images of parts of Fig.6A showing significant changes in the protein profiles. Arrows and circles indicate the relative positions of the spots in different lines.

Red blood cells (RBCs) are relatively simple cells that lack internal organelles and are enclosed by a plasma membrane. For iRBCs, the main parasite structure resides within a vacuolar membrane but progressively interacts with host proteins such as ankyrin and spectrin [36,37] in the RBC membrane by inserting parasite-derived proteins, forming knob-like structures and changing the rigidity of the infected cell [38]. In order to analyze parasite protein expression and distinguish free parasite protein from RBC membrane proteins, iRBCs were treated with hypotonic ionic buffer, to rupture and release their internal haemoglobin and free the parasite, leaving membrane ghosts that can be collected from the surface of the free-parasite pellet after centrifugation. This ghost fraction contains mainly erythrocyte membrane, as well as proteins from parasite source that are still associated with the erythrocyte membrane even after extensive washing. The free-parasite fraction more precisely reflects the expression of parasite proteins in general. When the 2D gel of RBC ghost fraction was stained with Coomassie Blue (Figure 1), A4 iRBC looked almost identical to 3D7 iRBC, with the only visible change being a 200 kilo Dalton (kDa) protein with different iso-electric point (PI), which was more acidic in A4 than in 3D7. This protein was identified as ankyrin by MALDI-TOF. As neither Coomassie Blue nor silver stain methods were able to highlight differential protein patterns between 3D7 and A4 isolates (Figure 1), [^35^S] methionine labelling *in vivo *was used so that only parasite proteins would be labelled and detected, as mature erythrocytes have no protein synthesis. As expected, the uninfected erythrocyte gel (data not shown) did not show any protein spots, while the iRBC ghost fraction contained a number of parasite proteins (Figures 2 and 3), with more parasite proteins seen in trophozoite than ring stage iRBC, suggesting that a number of parasite proteins have a tight association with the RBC membrane. The free-parasite fractions were also analysed after [^35^S] methionine labelling; the autoradiographs of the free parasite preparations of ring and trophozoite stages from three different parasite lines are shown in Figures 4 and 5. Consistent with the genotype identity of A4, C24 and ItG, their 2D protein maps show very similar protein profiles, with the small number of variable proteins indicated by circles on the gels. Nevertheless, since A4, C24 and ItG were selected *in vitro *for different adhesive phenotypes and are known to express variant PfEMP1 proteins, clone-specific protein expression profiles would be expected, particularly in proteins associated with the membrane or surface fraction. However, the autoradiographs of both iRBC erythrocyte membrane ghost and free-parasite fractions showed only very subtle changes. Using the 2D Evolution software, a number of changes were identified with over three-fold difference in signal intensity (see marked spots in Figures 2 to 5). Interestingly, most changes were between C24 and either A4 or ItG profiles, suggesting that A4 and ItG were more similar, consistent with their ability to bind ICAM-1. Another *P. falciparum *strain, 3D7, is a poorly cytoadherent line but the complete sequence of its genome makes it the best candidate for functional genomics and proteomics study. Therefore, 2D autoradiographs of parasites were compared from the IT4/25/5 lineage with 3D7. As shown in Figure 1, comparison of 3D7 with A4 did not reveal many changes, reflecting the predominance of host proteins. However, autoradiography revealed several distinctions between the protein profiles of the 3D7 trophozoite stage iRBC and either A4 or ItG (Figure 6A), while iRBC of A4 and ItG, as before, displayed almost identical maps. In the enlarged image of the boxed area of Figure 6A (Figure 6B), examples of protein position changes have been listed. In a limited area around pH4-5, molecular weight 45 to 60 kDa, three spots show significant changes from 3D7 and A4 or ItG, in all three cases resulting from more basic proteins in the 3D7 sample. This could be due to lower phosphorylation of these proteins in 3D7 than A4 or ItG, however, the role of phosphorylation in *P. falciparum *and its regulation are not known. The higher degree of difference in protein profiles between 3D7 and IT4/25/5-derived strains was also reflected in the comparison of profiles from ring stage iRBC, which also showed differences (data not shown). These changes were not seen on autoradiographs of the free-parasite fraction, suggesting that they could be membrane-associated events.

**Figure 7:**
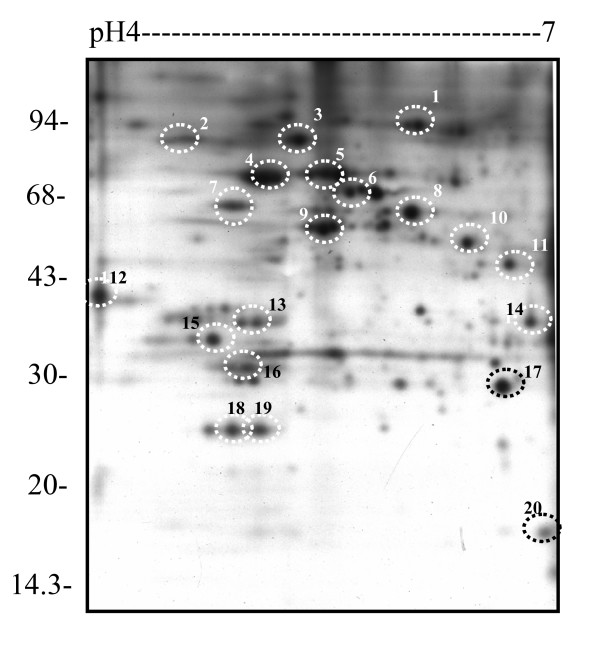
Identification of parasite proteins from A4 iRBC ghost fraction. The parasite proteins were traced by autoradiography, spots were excised and identified by MALDI-TOF. All marked protein IDs achieved statistically significant protein scores in the Mascot search results. An unannotated high quality psd file of the original gel is available (Additional file 20).

**Figure 8:**
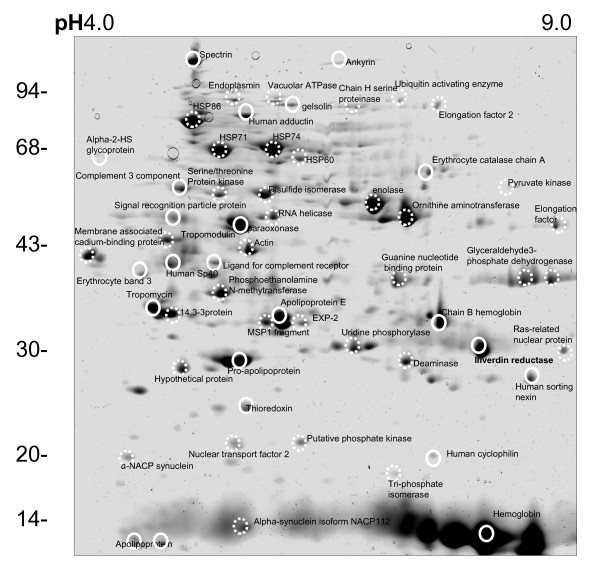
Representative 2D gel of parasite proteins from ItG trophozoites. ItG trophozoites were enriched 24 hours after invasion by Plasmagel floatation, the free parasite fraction was run on pH 3-10 IEF strips followed by 12.5% SDS-PAGE and the gel was stained with Coomassie Blue. Spots were excised and identified by MALDI-TOF. Solid circles indicate proteins from human source and dotted circles label proteins from parasite source. All marked protein IDs achieved significant protein scores in Mascot search results. An unannotated high quality tif file of the original gel is available (Additional file 21).

Autoradiography does not only describe the changes in parasite proteins but also can be used as an indicator to excise proteins for identification. As seen in Figure 7, radio-labelled iRBC samples showed over 100 proteins of parasite origin in the iRBC membrane fraction. When this type of gel is stained with Coomassie Blue, the most abundant proteins identified by MALDI-TOF were from human source. Therefore, the positions of spots on the autoradiograph were used to trace parasite proteins. Since [^35^S] methionine is a low energy isotope, the X-ray film needs to be in direct contact with the 2D gels, so the gel has to be dried, necessitating protein identification on dry spots. Despite these technical difficulties, identified a number of parasite proteins were identified (Table 1). However, there remain difficulties in identifying all the proteins, particularly low abundant ones. Indeed a number of well-characterized parasite proteins known to be involved in membrane association or surface localization (e.g. PfEMP1) could not be seen from the 2D map, most likely because of low abundance and hydrophobic nature, setting them out of the range of the protein purification and separation methodologies used here.

**Table 1:** Protein identifications (see Fig. 7)

Number	Protein ID	Accession No.	M (KDa)	PI	No. of peptides matched	% of SQ rec	Mass score
1	Putative HSP90	Gi 23508379	109.195	6.22	22	26	74
2	Glycophorin-binding protein 130 precursor	Gi121507	90.077	5.02	14	35	99
3	HSP86	Gi23612467	86.512	4.95	10	25	87
4	78KD glucose regulated protein	Gi 1170013	72.845	5.38	21	35	89
5	78KD glucose regulated protein	Gi 1170013	72.845	5.38	21	35	89
6	PFhsp70-3	Gi 11127605	71.945	5.30	7	13	81
7	Hypothetical protein	Gi 23613336	60720	4.95	14	21	48
8	HSP60	Gi 23507957	62.911	6.75	14	23	59
9	Protein disulfide isomerase	Gi 11125364	55.782	5.57	8	26	78
10	Enolase	Gi 3023709	49.015	6.21	10	29	91
11	EF-1alpha	Gi 9887	49.238	9.27	12	32	72
12	Endoplasmic reticulum-resident calcium binding protein	Gi 23508293	39.464	4.0	18	45	143
13	Variant surface antigen rifin	Gi 23505091	40.869	8.72	11	30	84
14	RESA-like proteinTruncated	Gi 23510225	36.224	6.7	6	21	43 +psd
15	Phosphoethanolamine N-methyltransferase	Gi 23619361	31.309	5.43	11	38	74
16	Falcipain	Gi76257570	27.623	4.96	8	45	61
17	Uridine phosphorylase	Gi 23613155	27.525	6.27	11	52	141
18	Hypothetical protein	Gi 23613776	24.911	5.49	11	36	84
19	Hypothetical protein	Gi 23613776	24.911	5.49	11	36	84
20	Peptidyl-prolyl cis-trans isomerase	Gi 23508355	21.831	7.10	7	30	57 +psd

Parasite proteins only account for a small amount of total infected erythrocyte proteins, with over 70% of the protein spots identified as human proteins (data not shown). To further extract parasite proteins for identification, parasites were synchronized by Plasmagel flottation to enrich trophozoite stage parasites up to 80%, treated infected erythrocytes with saponin for less than 7 min and then washed the free-parasite fraction extensively. The brief saponin treatment produces large pieces of erythrocyte membrane and free parasite, repeated washing with ice cold saline solution removes most of the contaminating host erythrocyte lysate and, therefore, enriches for parasite proteins. This sample was separated by 2D gel electrophoresis (Figure 8) and using MALDI-TOF, 58 proteins were identified from this gel, of which 28 came from parasite source.

A number of techniques can be used to increase the rate of protein identification and try to find the surface protein specifically. For example, Multidimensional Protein Identification Technology (MudPIT), a two-dimensional liquid chromatograph coupled with tandem mass spectrometry [39]; molecules exposed on the iRBC surface can be digested by enzyme treatment such as trypsin or chymotrypsin [40,41]; insertion molecules can be purified from membrane fractions [42]. Yimin Wu's group used biotin-streptavidin labeling to purify all proteins on the iRBC surface and identified proteins by shotgun proteomic analysis [43]. Such techniques certainly provide high throughput scale and improve the identification sensitivity. However, a problem still remained as some cytosol proteins were also present in the pool of surface proteins because of internal biotin labelling and high abundant protein non-specifically binding to streptavidin. Recently, Lingelbach's group has used furosemide, a non-specific anion transport inhibitor, to block parasite-induced biotin permeability, making biotin-streptavidin technique more specific [44]. Another way to trace protein trafficking or protein remodelling or protein targeting is by *in silico *prediction of host-targeting signals required for the export of proteins from the parasite vacuole to the erythrocyte membrane [22,23].

The predominant modification seen on 2D gels was horizontal position changes rather than presence or absence of a protein, indicating protein post-translational modification such as phosphorylation, which increases the negative charge of the protein and shifts the iso-electric point (PI) to the more acidic side. In yeast, protein phosphorylation is estimated to affect 30% of the proteome. Using proteome chip technology, scientists have determined the *in vitro *substrates recognized by yeast protein kinases. 4,192 phosphorylation events involving 1,325 different proteins were identified and kinase-substrate relationships distinguished [45]. The results suggest that host and parasite proteins may experience phosphorylation changes during parasite development inside the erythrocytes.

## Conclusion

Using a sensitive *in vivo *labelling technique allied to 2D electrophoresis, highly reproducible, limited changes in *P. falciparum *protein profiles from a number of parasite lines have been shown, differing in their adhesive phenotypes, with even fewer differences seen in antigenically distinct clones sharing the ability to bind to ICAM-1. Of the changes detected, most were alterations in PI, indicative of differential phosphorylation. Only a subset of the protein spots could be identified using mass spectrometry. Other studies have used non-gel methodologies, such as nano-LC/MS/MS to overcome this problem, but these also have limitations for low abundance, membrane proteins and several proteins known to be at the surface of the infected erythrocyte were not identified by this approach. Further development of biochemical techniques, detection systems and sequence databases, particularly for variant surface antigen families, will be required to identify the full complement of parasite proteins in the infected erythrocyte membrane.

## Authors' contributions

Both authors participated in the design of the study. YW carried out all the experimental work. AC conceived of the study, participated in its coordination and helped to draft the manuscript. Both authors read and approved the final manuscript

## Supplementary Material

Additional file 1 2D electrophoresis profile of iRBC ghosts from 3D7 stained with Coomassie Blue (see figure 1). Click here for file

Additional file 2 2D electrophoresis profile of iRBC ghosts from 3D7 stained with silver (see figure 1). Click here for file

Additional file 3 2D electrophoresis profile of iRBC ghosts from A4 stained with Coomassie Blue (see figure 1). Click here for file

Additional file 4 2D electrophoresis profile of iRBC ghosts from A4 stained with silver (see figure 1). Click here for file

Additional file 5 Autoradiograph of 2D electrophoresis profile of metabolically labelled proteins from ring stage iRBC ghosts from A4 (see figure 2). Click here for file

Additional file 6 Autoradiograph of 2D electrophoresis profile of metabolically labelled proteins from ring stage iRBC ghosts from C24 (see figure 2). Click here for file

Additional file 7 Autoradiograph of 2D electrophoresis profile of metabolically labelled proteins from ring stage iRBC ghosts from ItG (see figure 2). Click here for file

Additional file 8 Autoradiograph of 2D electrophoresis profile of metabolically labelled proteins from trophozoite stage iRBC ghosts from A4 (see figure 3). Click here for file

Additional file 9 Autoradiograph of 2D electrophoresis profile of metabolically labelled proteins from trophozoite stage iRBC ghosts from C24 (see figure 3). Click here for file

Additional file 10 Autoradiograph of 2D electrophoresis profile of metabolically labelled proteins from trophozoite stage iRBC ghosts from ItG (see figure 3). Click here for file

Additional file 11 Autoradiograph of 2D electrophoresis profile of metabolically labelled proteins from ring stage free parasites from A4 (see figure 4). Click here for file

Additional file 12 Autoradiograph of 2D electrophoresis profile of metabolically labelled proteins from ring stage free parasites from C24 (see figure 4). Click here for file

Additional file 13 Autoradiograph of 2D electrophoresis profile of metabolically labelled proteins from ring stage free parasites from ItG (see figure 4). Click here for file

Additional file 14 Autoradiograph of 2D electrophoresis profile of metabolically labelled proteins from trophozoite stage free parasites from A4 (see figure 5). Click here for file

Additional file 15 Autoradiograph of 2D electrophoresis profile of metabolically labelled proteins from trophozoite stage free parasites from C24 (see figure 5). Click here for file

Additional file 16 Autoradiograph of 2D electrophoresis profile of metabolically labelled proteins from trophozoite stage free parasites from ItG (see figure 5). Click here for file

Additional file 17 Autoradiograph of 2D electrophoresis profile of metabolically labelled proteins from iRBC ghosts from 3D7 (see figure 6). Click here for file

Additional file 18 Autoradiograph of 2D electrophoresis profile of metabolically labelled proteins from iRBC ghosts from A4 (see figure 6). Click here for file

Additional file 19 Autoradiograph of 2D electrophoresis profile of metabolically labelled proteins from iRBC ghosts from ItG (see figure 6). Click here for file

Additional file 20 Preparative 2D gel for protein identification of A4 iRBC ghost fraction (see figure 7 and table 1). Click here for file

Additional file 21 Unannotated 2D electrophoresis profile from ItG trophozoite, free parasite fraction (see figure 8). Click here for file
