# Supplementary figures and images for: Comparative proteomic analysis of metabolically labelled proteins from Plasmodium falciparum isolates with different adhesion properties
Source: Malar J. 2006 Aug 3;5:67. doi: 10.1186/1475-2875-5-67 (PMC1559632; doi:10.1186/1475-2875-5-67)

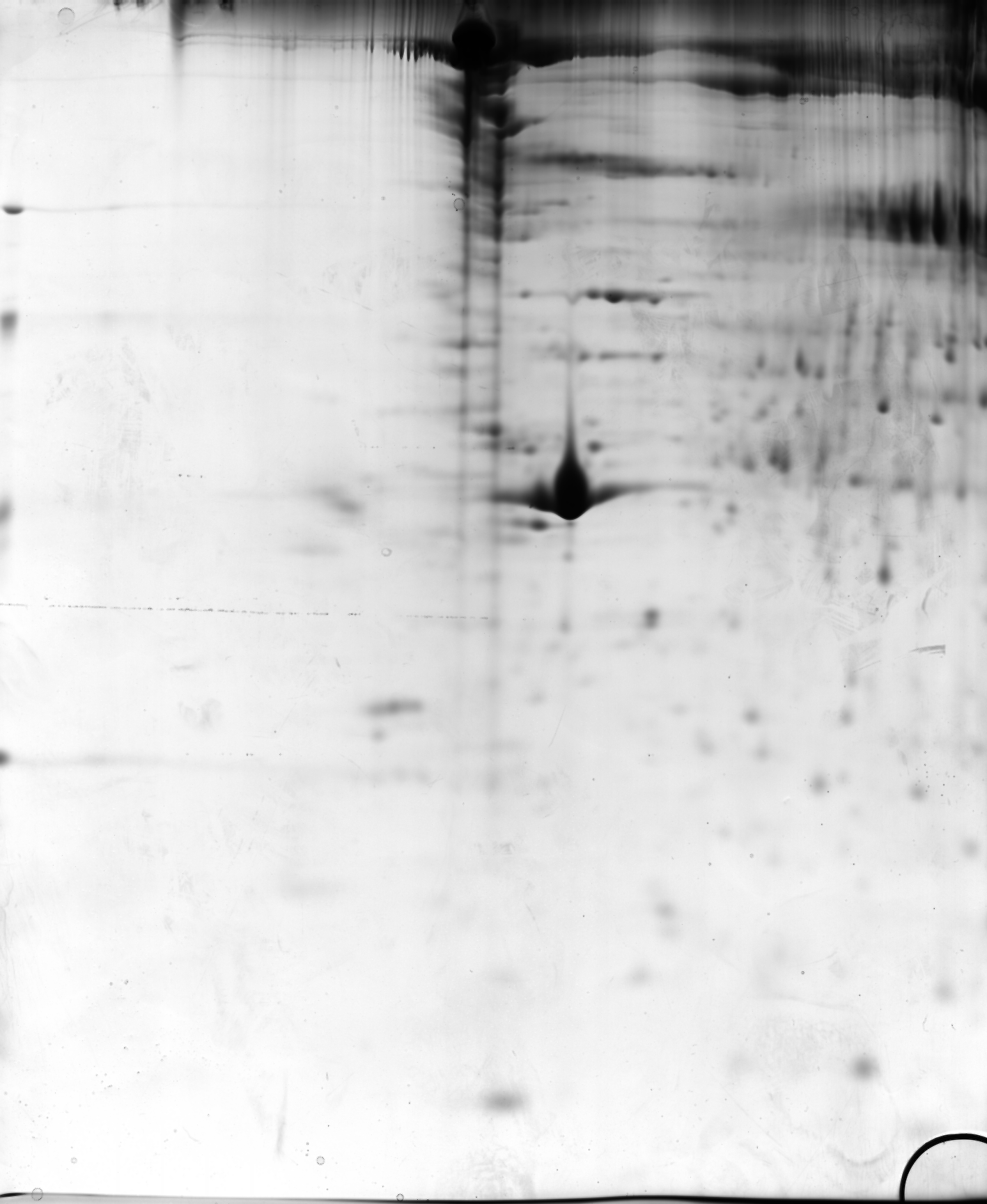

Supplement: Additional file 1 — 2D electrophoresis profile of iRBC ghosts from 3D7 stained with Coomassie Blue (see figure 1). [file 1475-2875-5-67-S1.tiff]

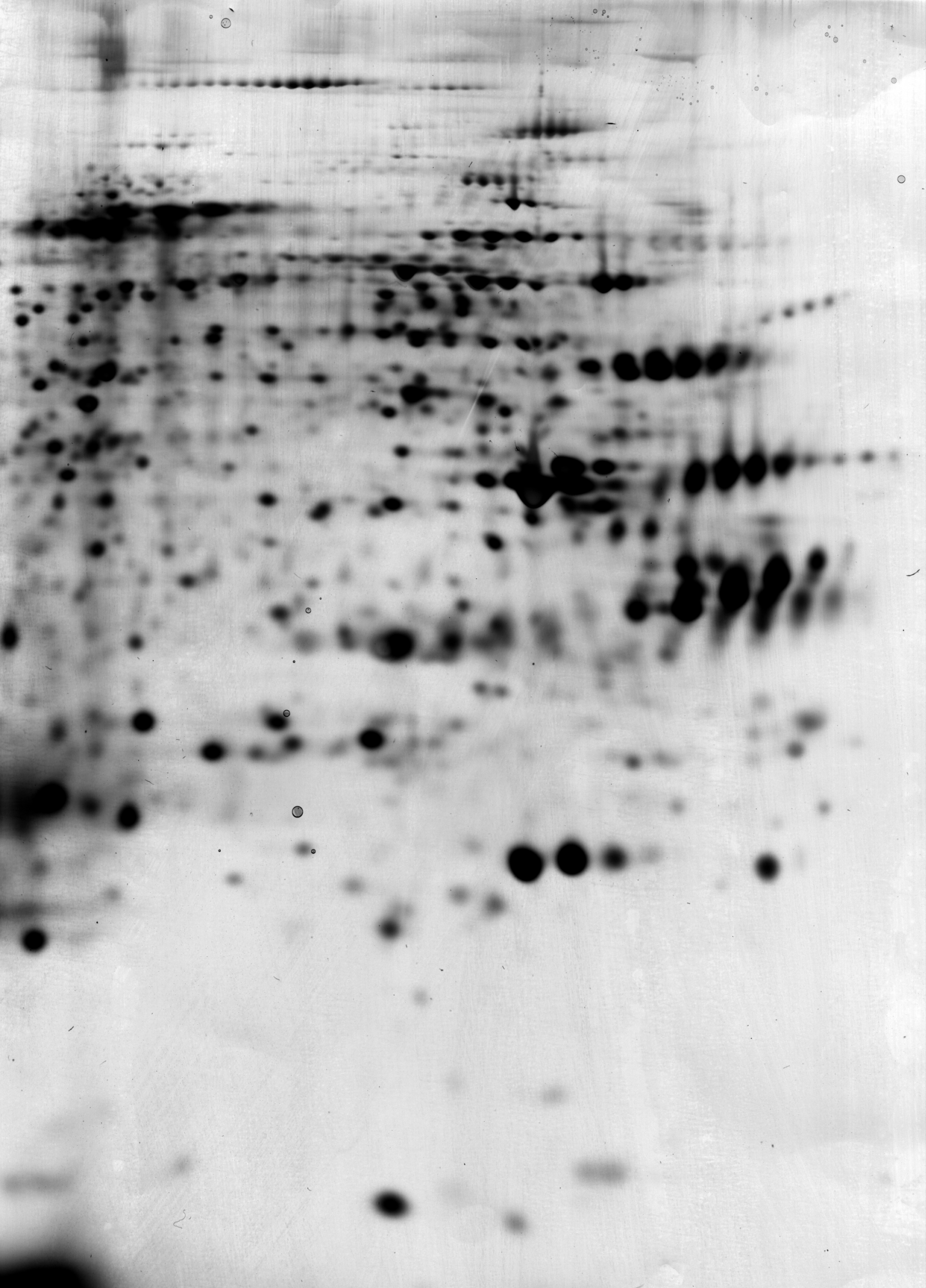

Supplement: Additional file 2 — 2D electrophoresis profile of iRBC ghosts from 3D7 stained with silver (see figure 1). [file 1475-2875-5-67-S2.tiff]

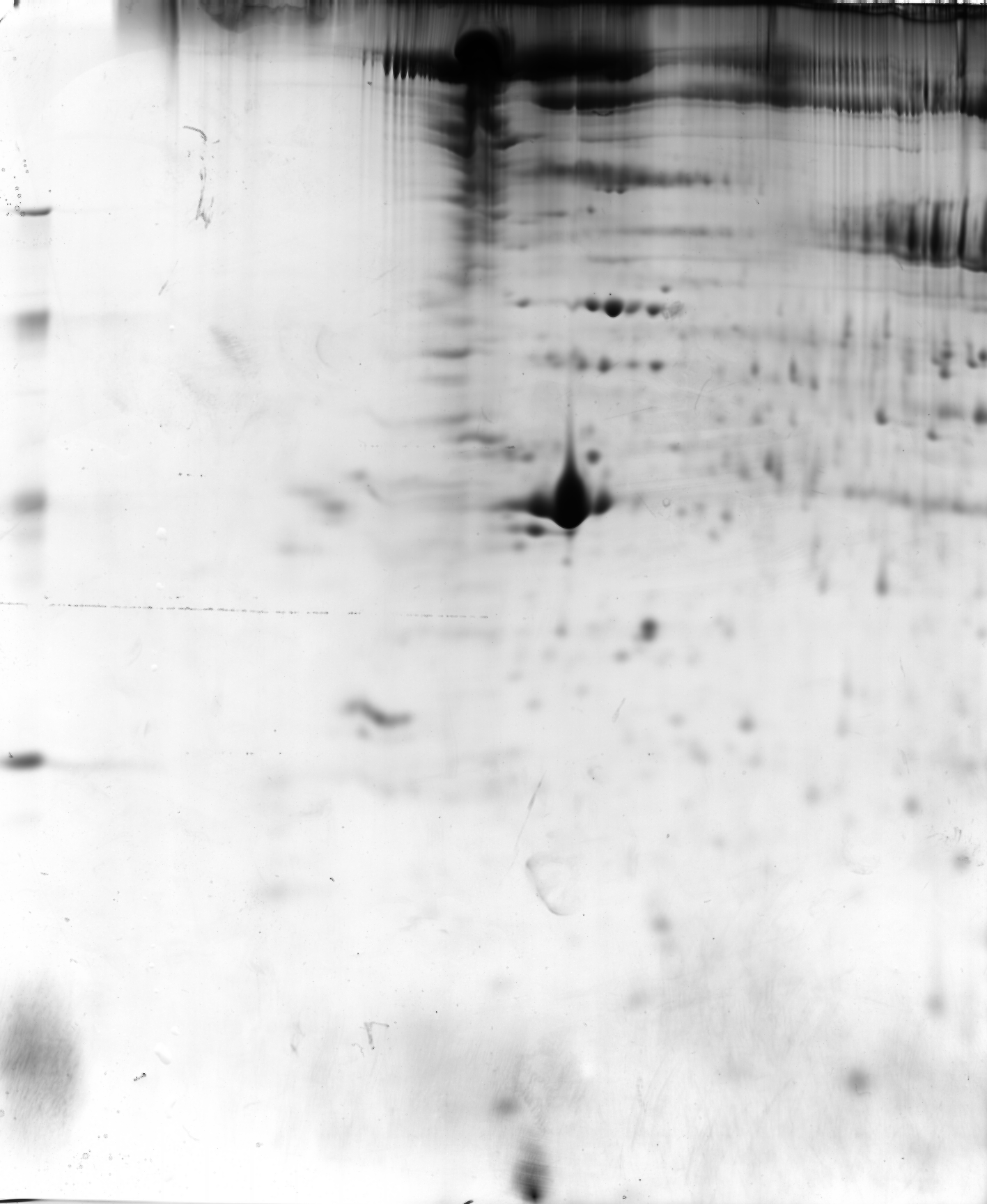

Supplement: Additional file 3 — 2D electrophoresis profile of iRBC ghosts from A4 stained with Coomassie Blue (see figure 1). [file 1475-2875-5-67-S3.tiff]

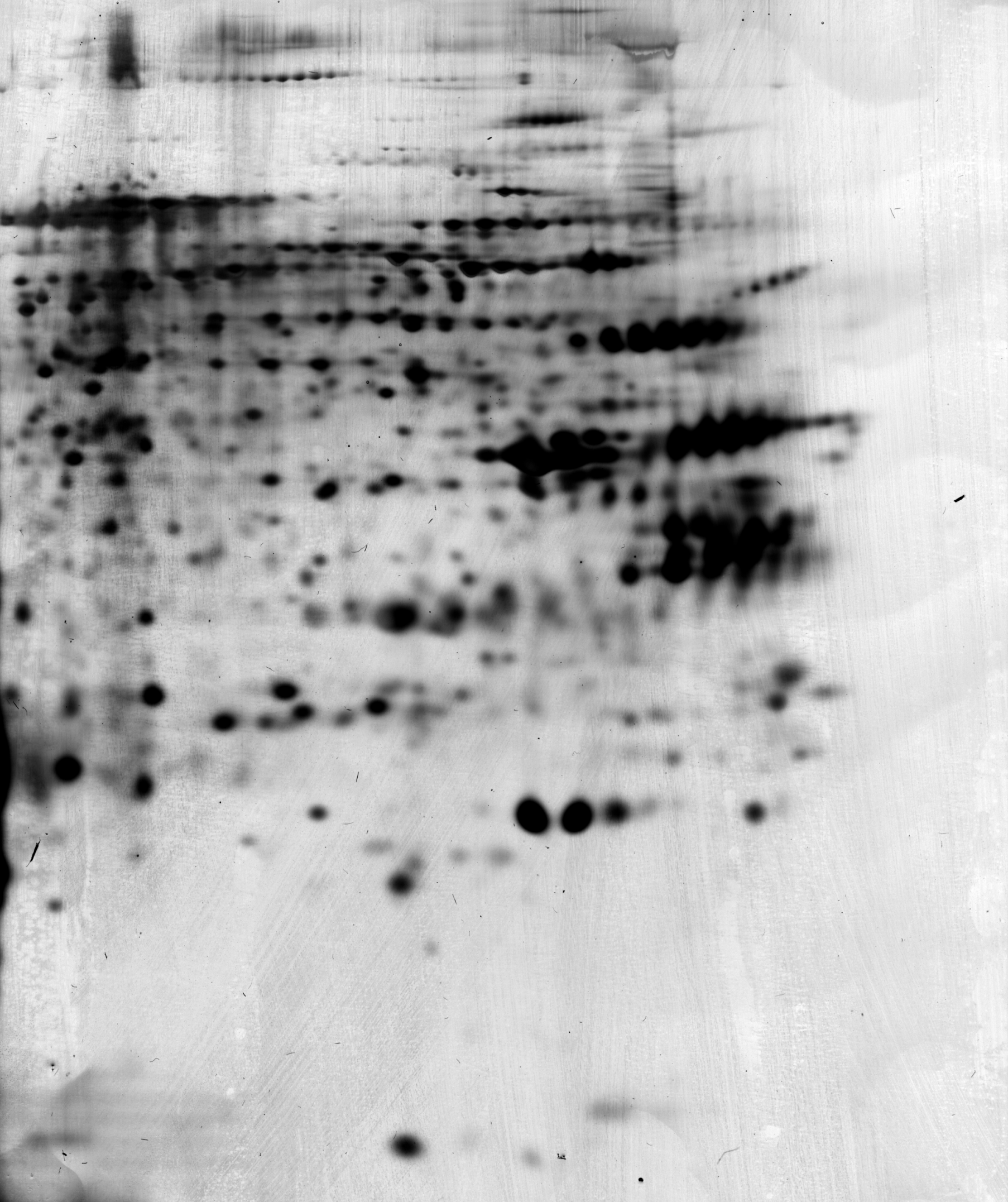

Supplement: Additional file 4 — 2D electrophoresis profile of iRBC ghosts from A4 stained with silver (see figure 1). [file 1475-2875-5-67-S4.tiff]

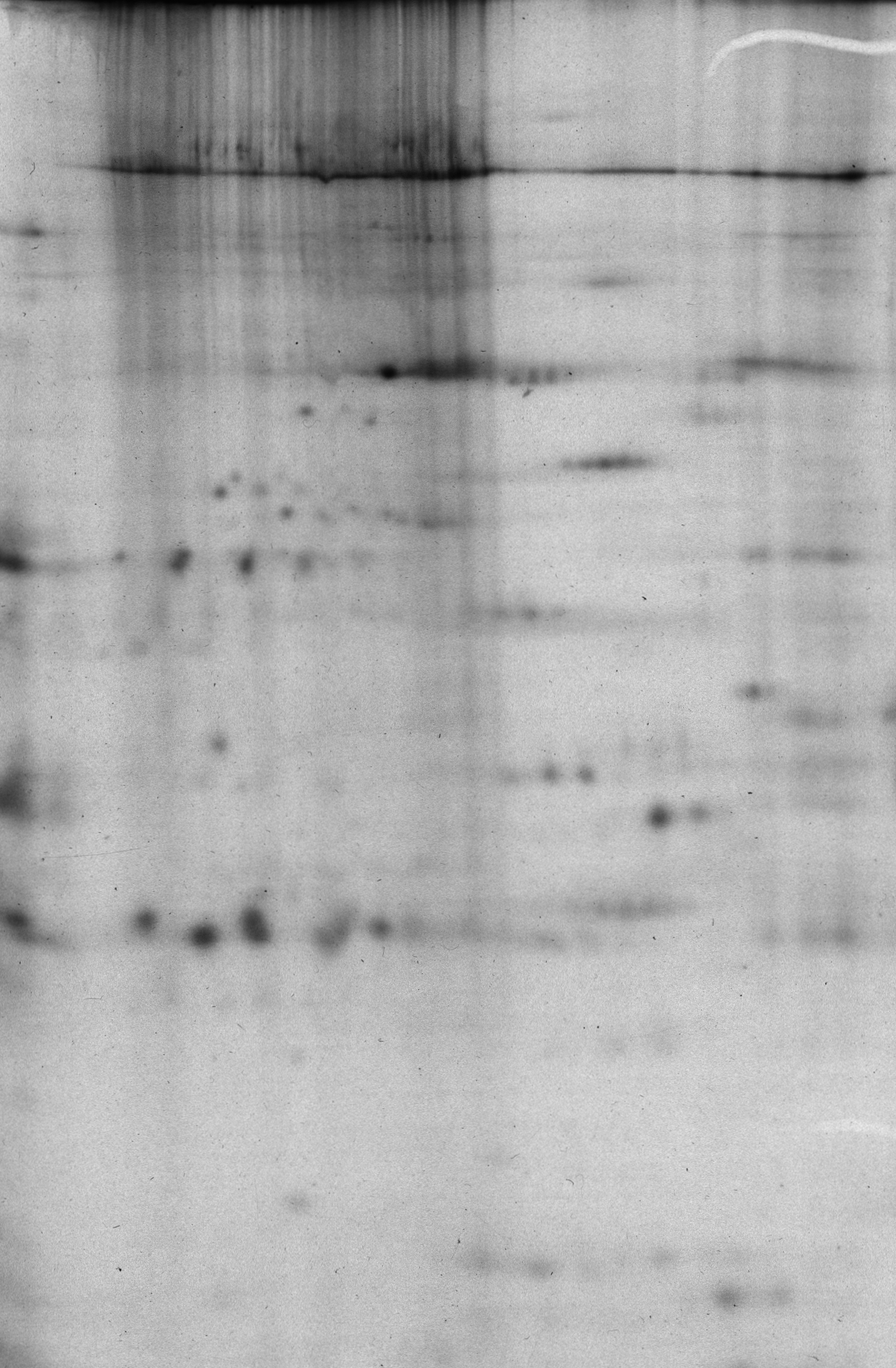

Supplement: Additional file 5 — Autoradiograph of 2D electrophoresis profile of metabolically labelled proteins from ring stage iRBC ghosts from A4 (see figure 2). [file 1475-2875-5-67-S5.tiff]

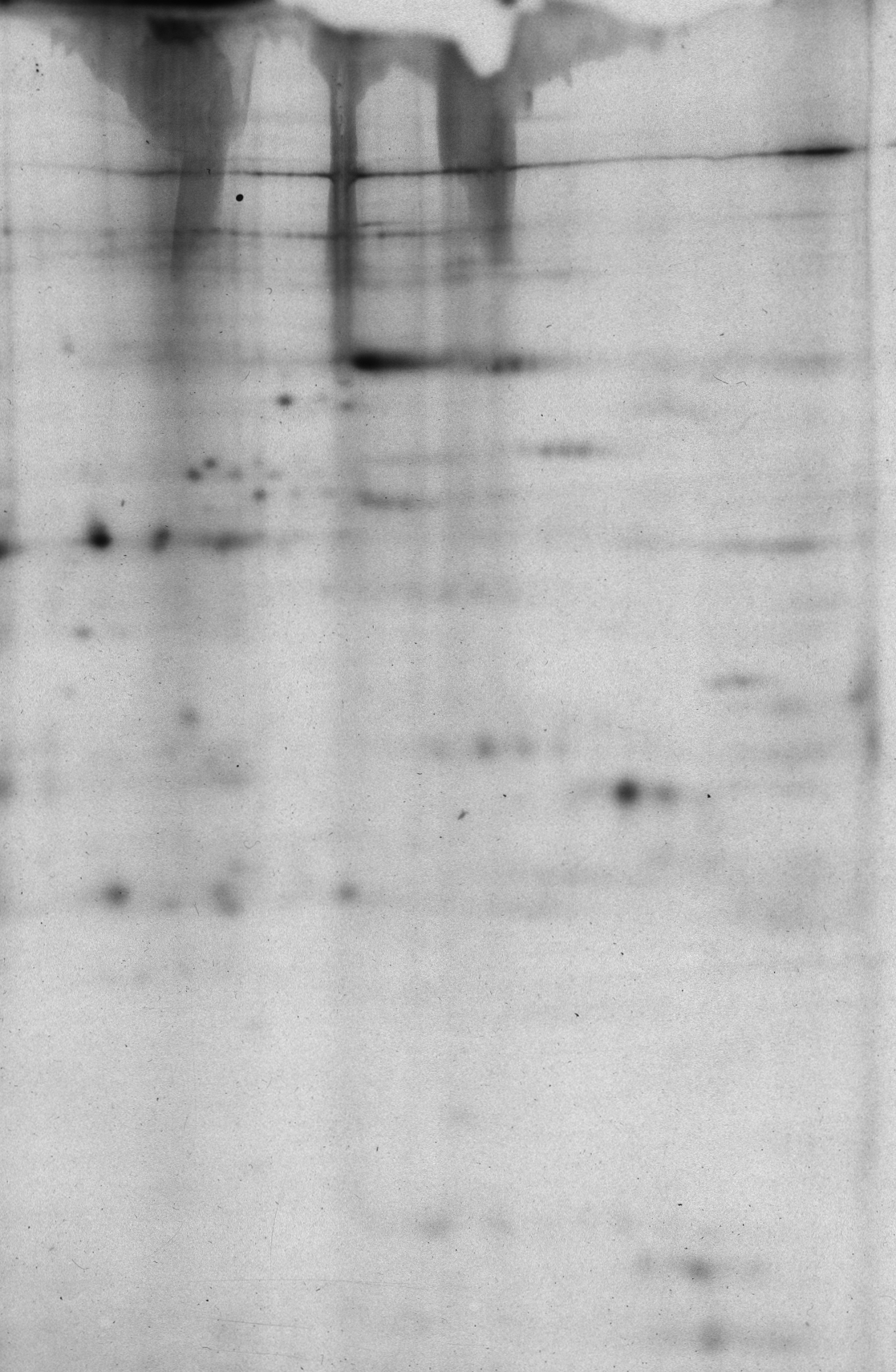

Supplement: Additional file 6 — Autoradiograph of 2D electrophoresis profile of metabolically labelled proteins from ring stage iRBC ghosts from C24 (see figure 2). [file 1475-2875-5-67-S6.tiff]

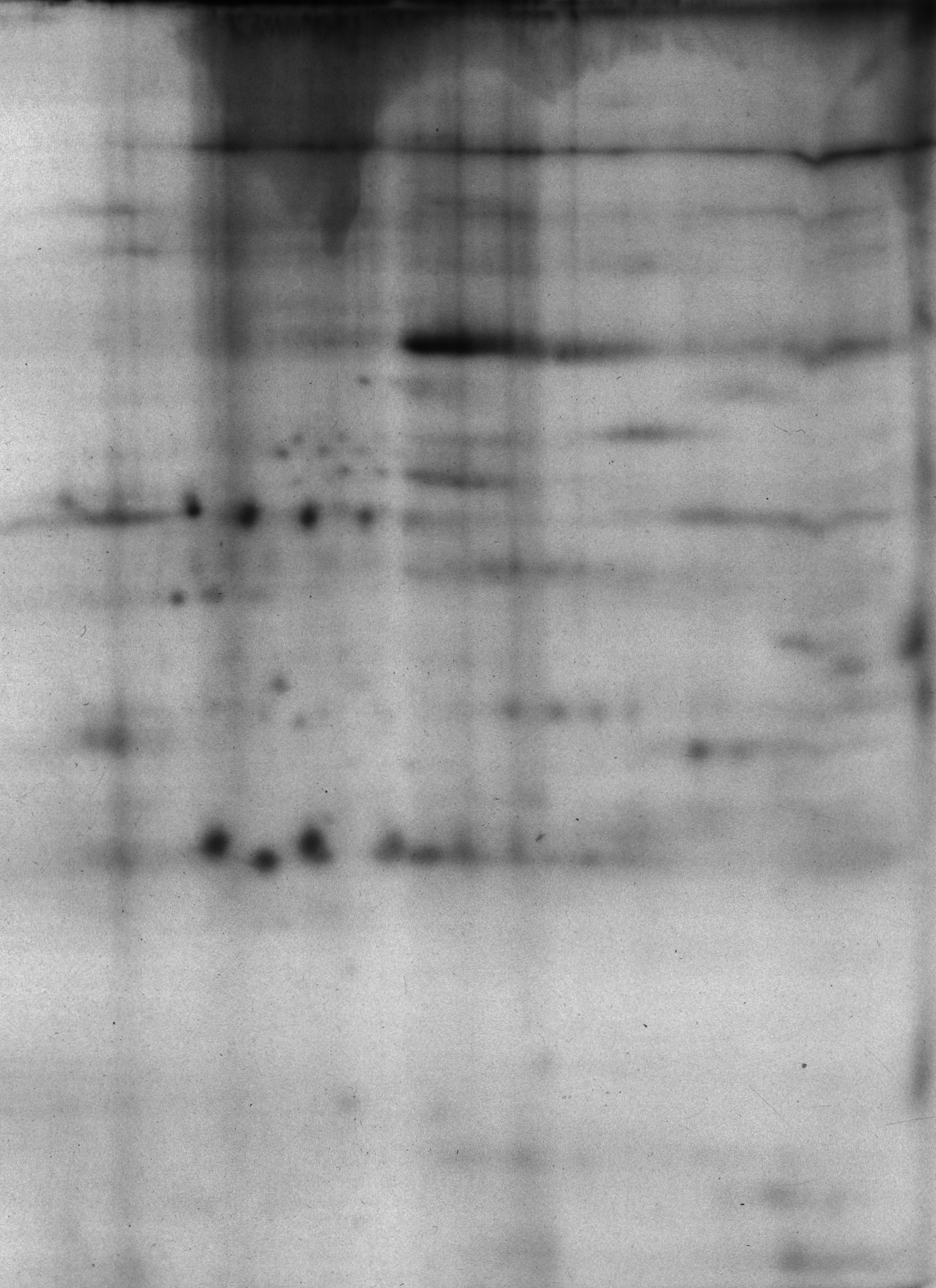

Supplement: Additional file 7 — Autoradiograph of 2D electrophoresis profile of metabolically labelled proteins from ring stage iRBC ghosts from ItG (see figure 2). [file 1475-2875-5-67-S7.tiff]

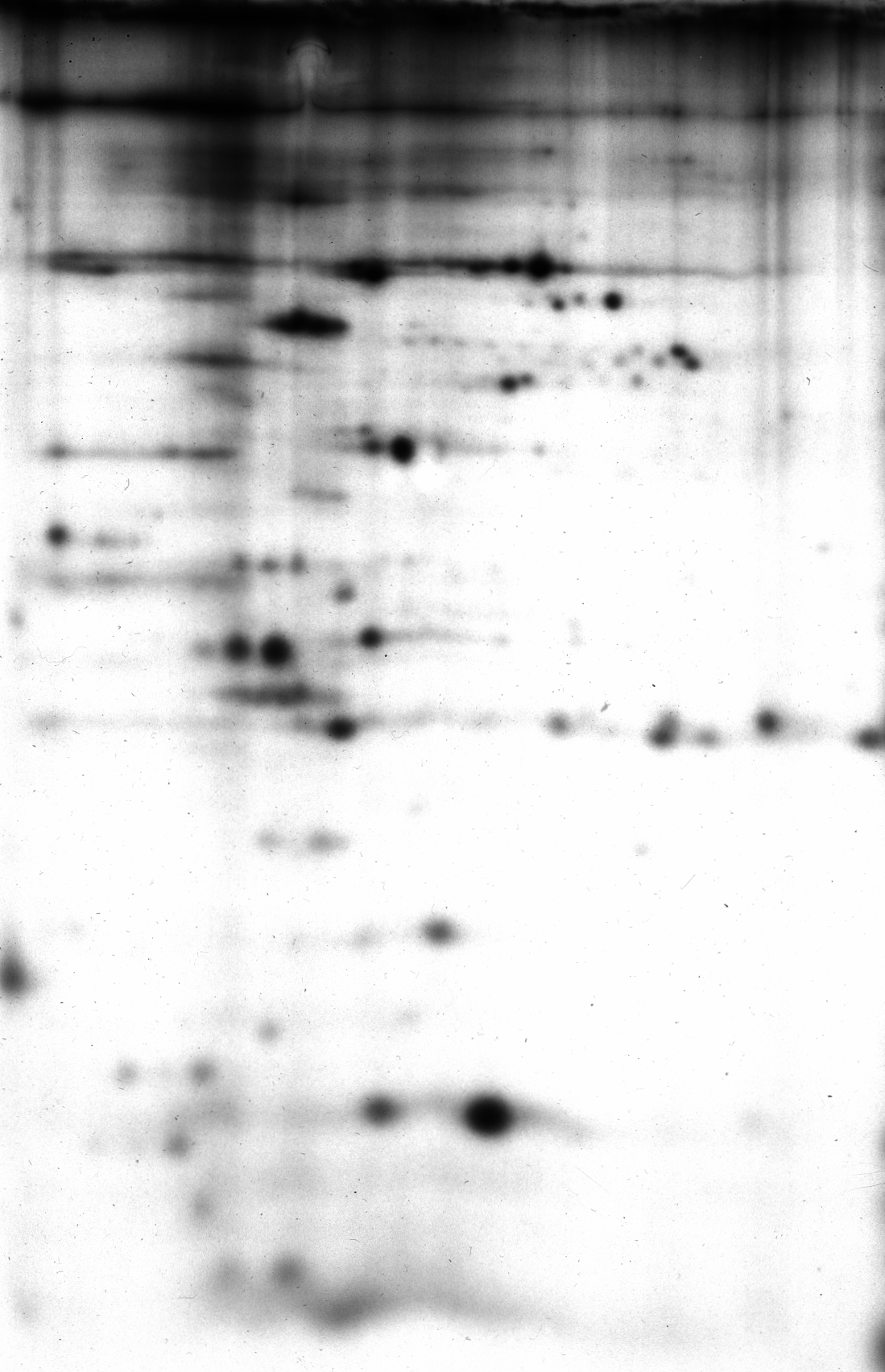

Supplement: Additional file 8 — Autoradiograph of 2D electrophoresis profile of metabolically labelled proteins from trophozoite stage iRBC ghosts from A4 (see figure 3). [file 1475-2875-5-67-S8.tiff]

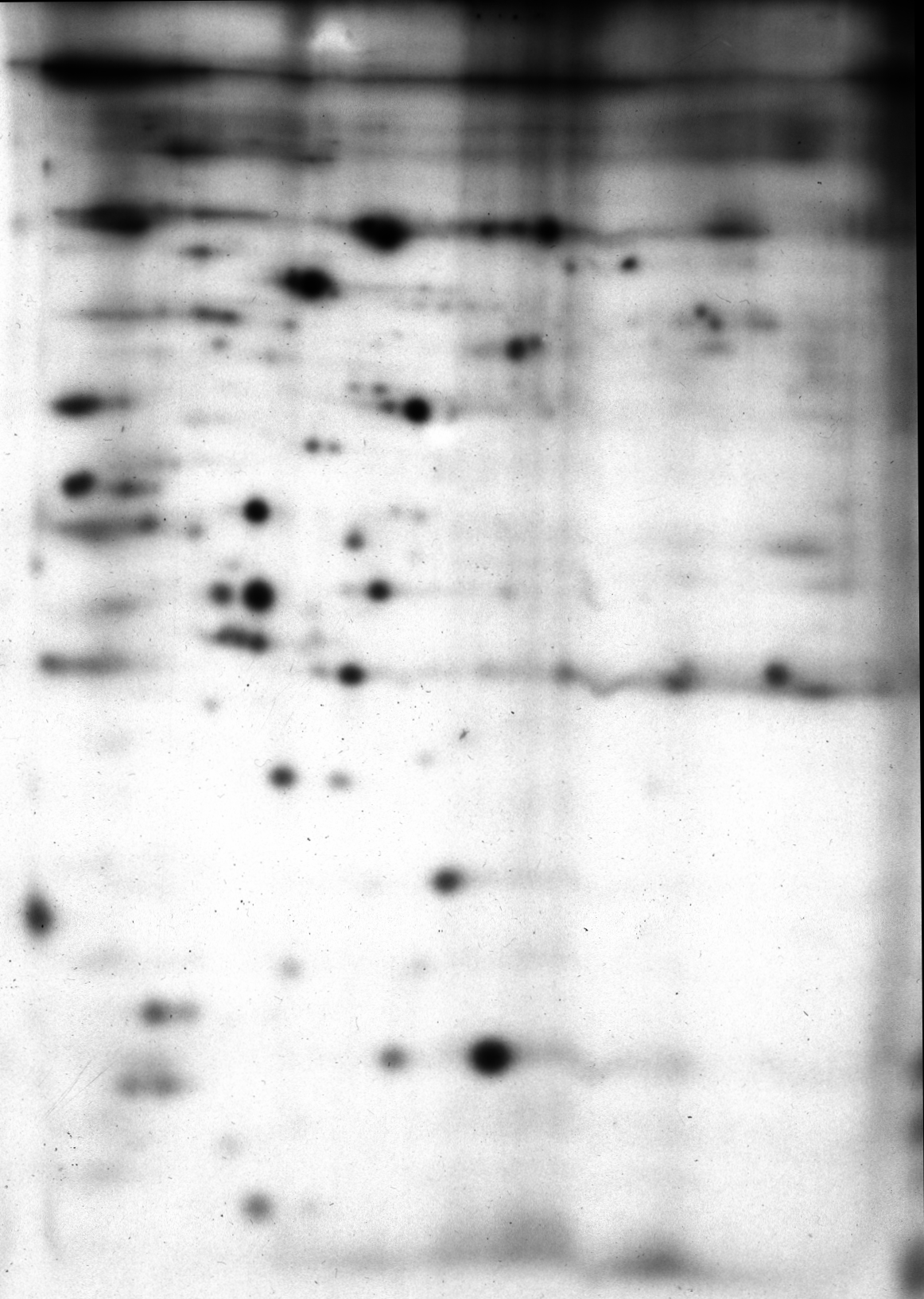

Supplement: Additional file 9 — Autoradiograph of 2D electrophoresis profile of metabolically labelled proteins from trophozoite stage iRBC ghosts from C24 (see figure 3). [file 1475-2875-5-67-S9.tiff]

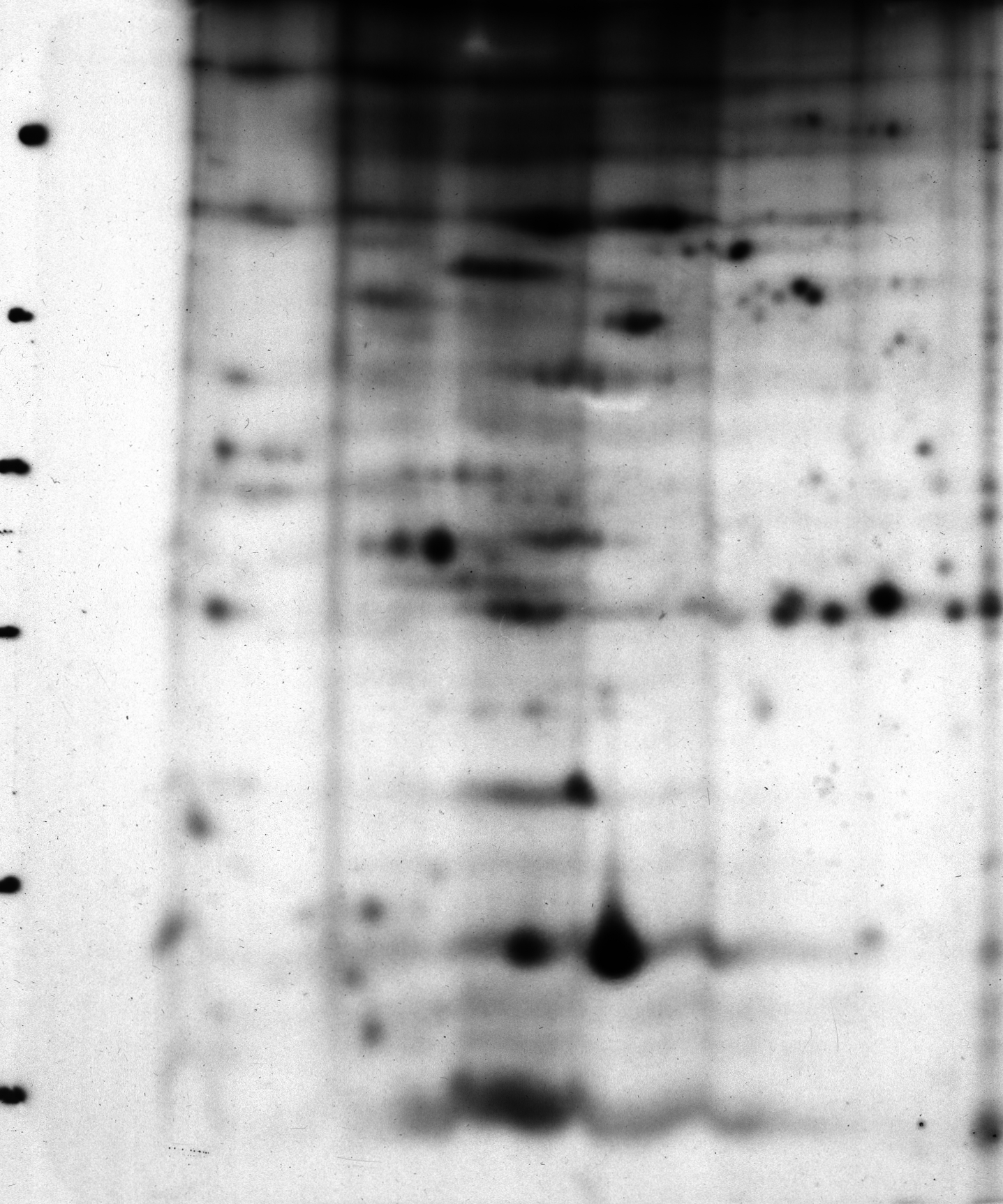

Supplement: Additional file 10 — Autoradiograph of 2D electrophoresis profile of metabolically labelled proteins from trophozoite stage iRBC ghosts from ItG (see figure 3). [file 1475-2875-5-67-S10.tiff]

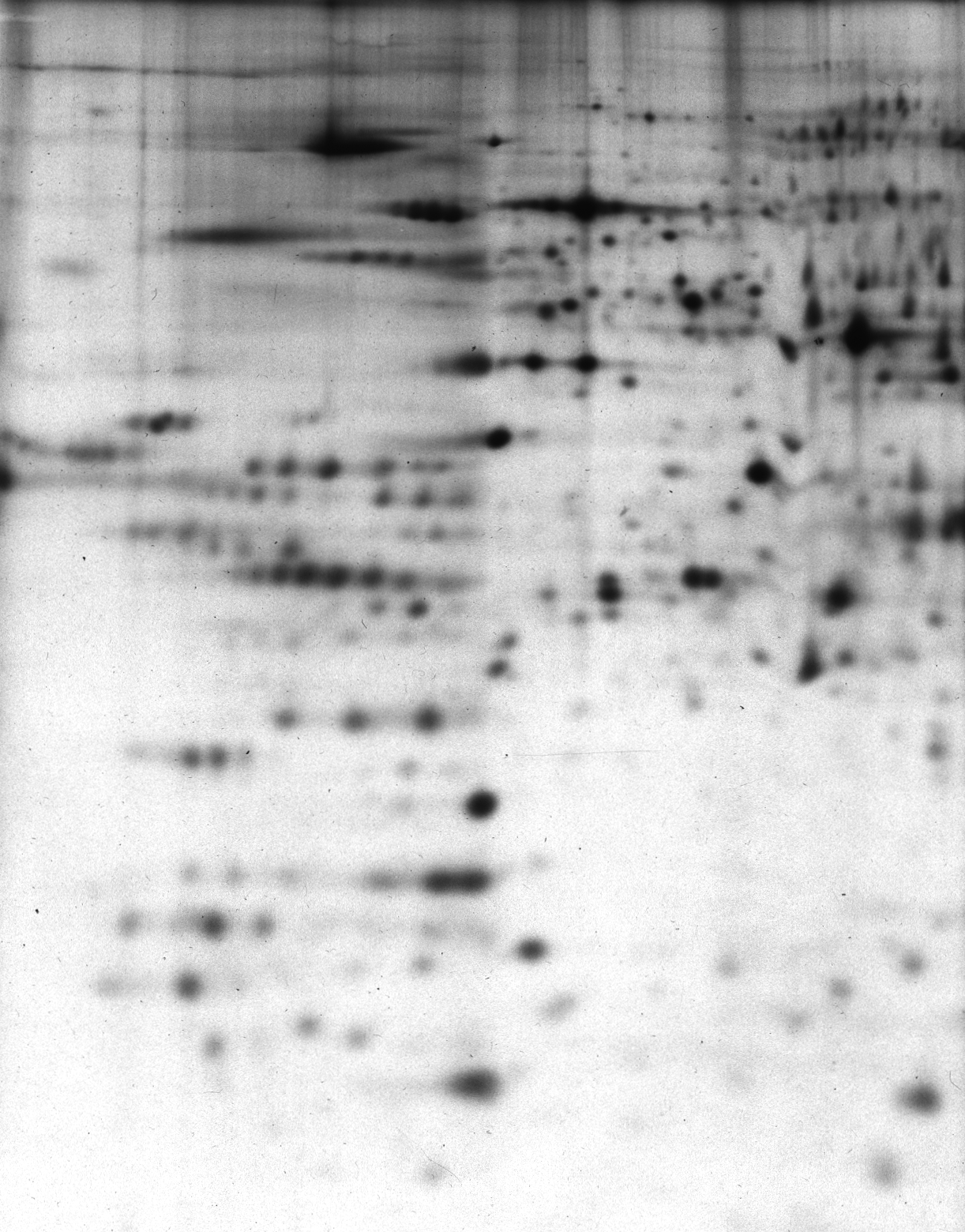

Supplement: Additional file 11 — Autoradiograph of 2D electrophoresis profile of metabolically labelled proteins from ring stage free parasites from A4 (see figure 4). [file 1475-2875-5-67-S11.tiff]

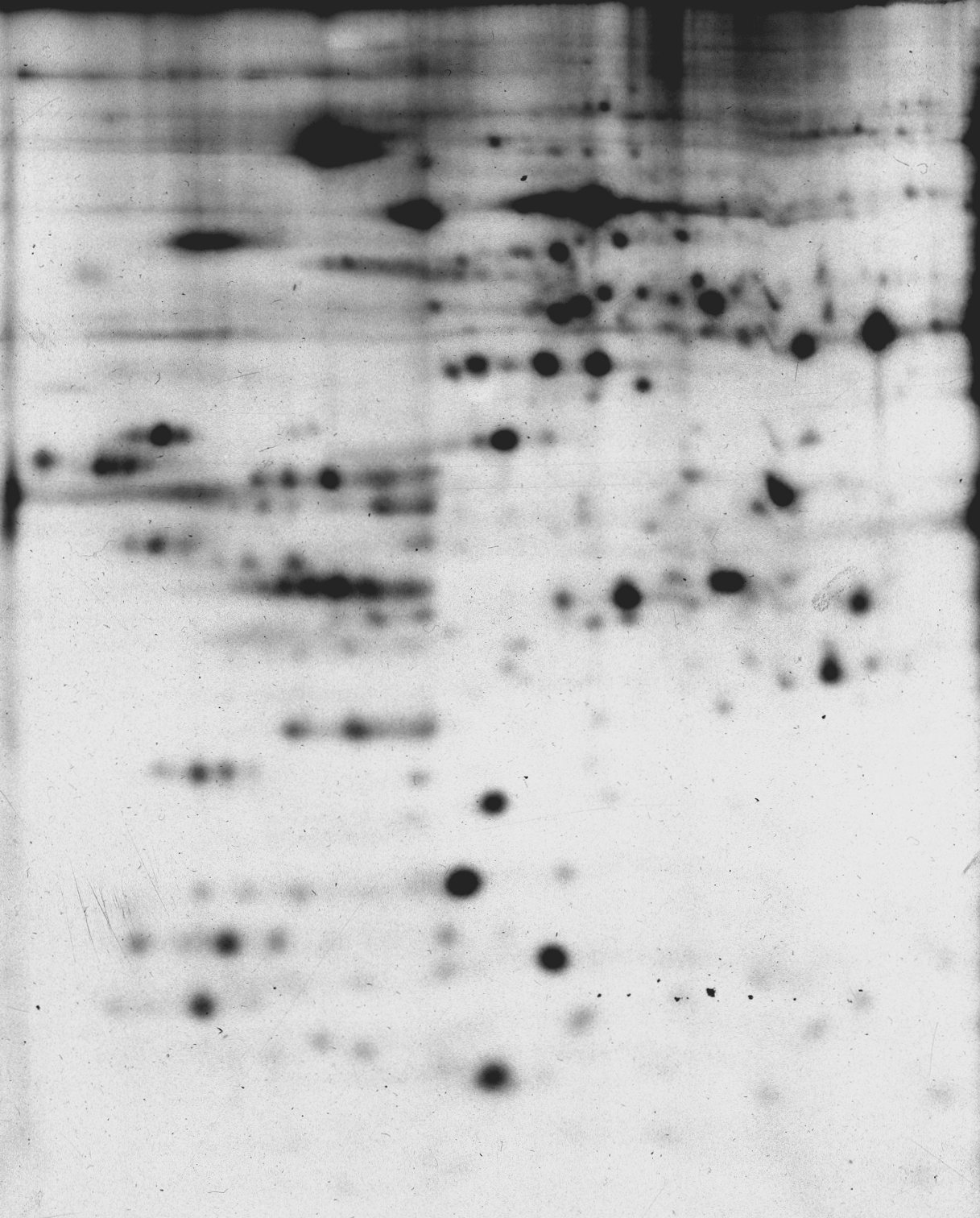

Supplement: Additional file 12 — Autoradiograph of 2D electrophoresis profile of metabolically labelled proteins from ring stage free parasites from C24 (see figure 4). [file 1475-2875-5-67-S12.tiff]

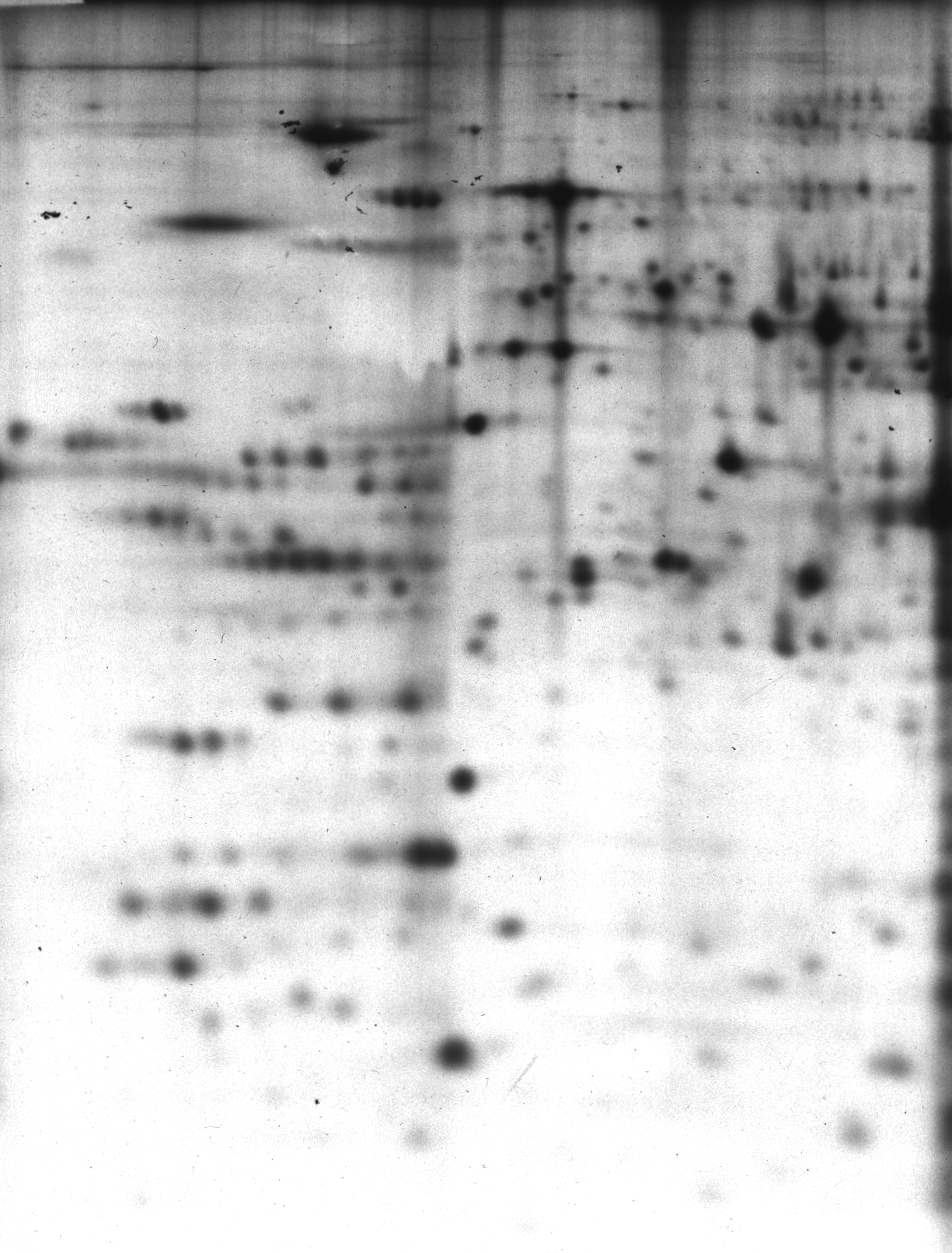

Supplement: Additional file 13 — Autoradiograph of 2D electrophoresis profile of metabolically labelled proteins from ring stage free parasites from ItG (see figure 4). [file 1475-2875-5-67-S13.tiff]

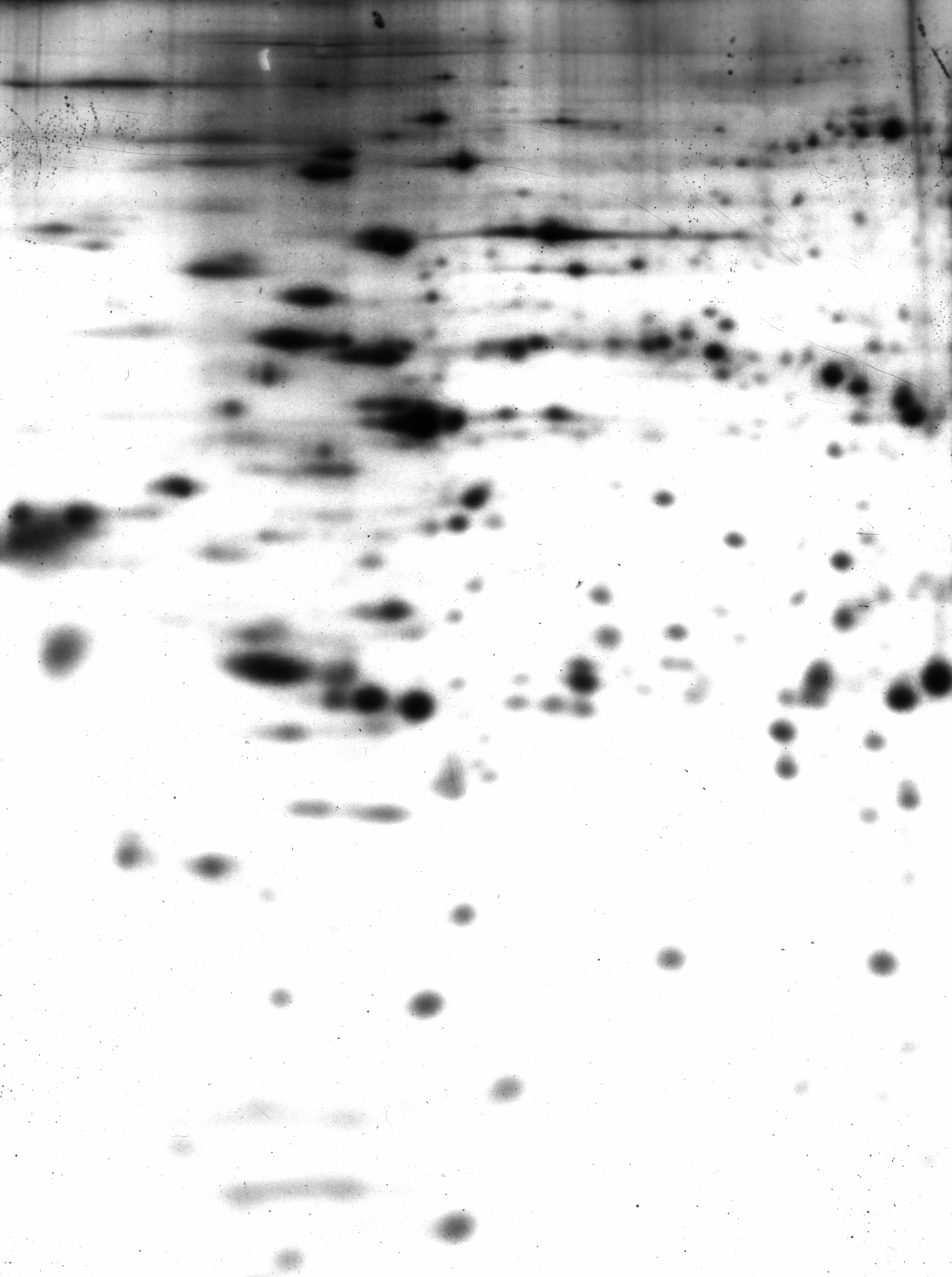

Supplement: Additional file 14 — Autoradiograph of 2D electrophoresis profile of metabolically labelled proteins from trophozoite stage free parasites from A4 (see figure 5). [file 1475-2875-5-67-S14.tiff]

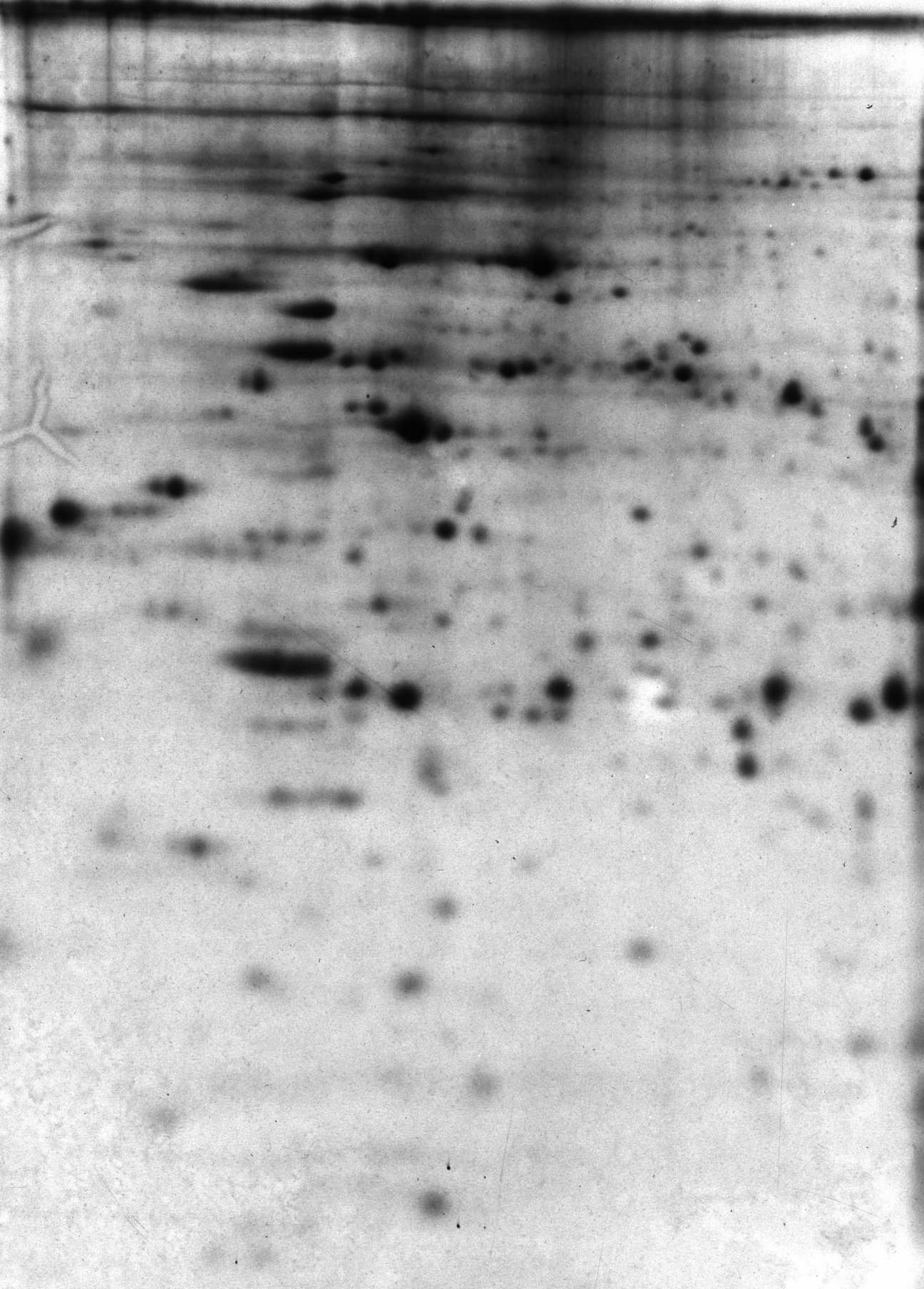

Supplement: Additional file 15 — Autoradiograph of 2D electrophoresis profile of metabolically labelled proteins from trophozoite stage free parasites from C24 (see figure 5). [file 1475-2875-5-67-S15.tiff]

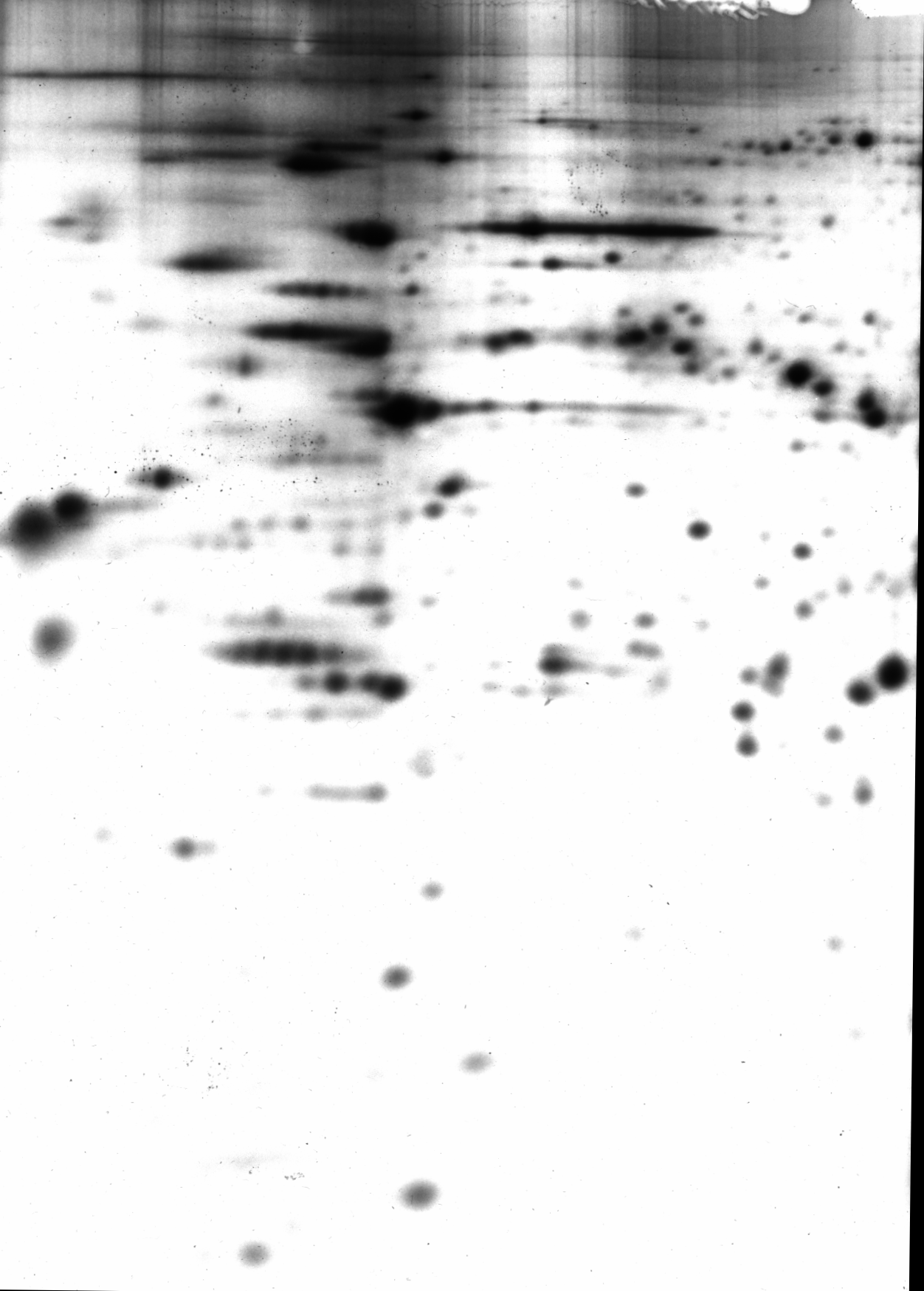

Supplement: Additional file 16 — Autoradiograph of 2D electrophoresis profile of metabolically labelled proteins from trophozoite stage free parasites from ItG (see figure 5). [file 1475-2875-5-67-S16.tiff]

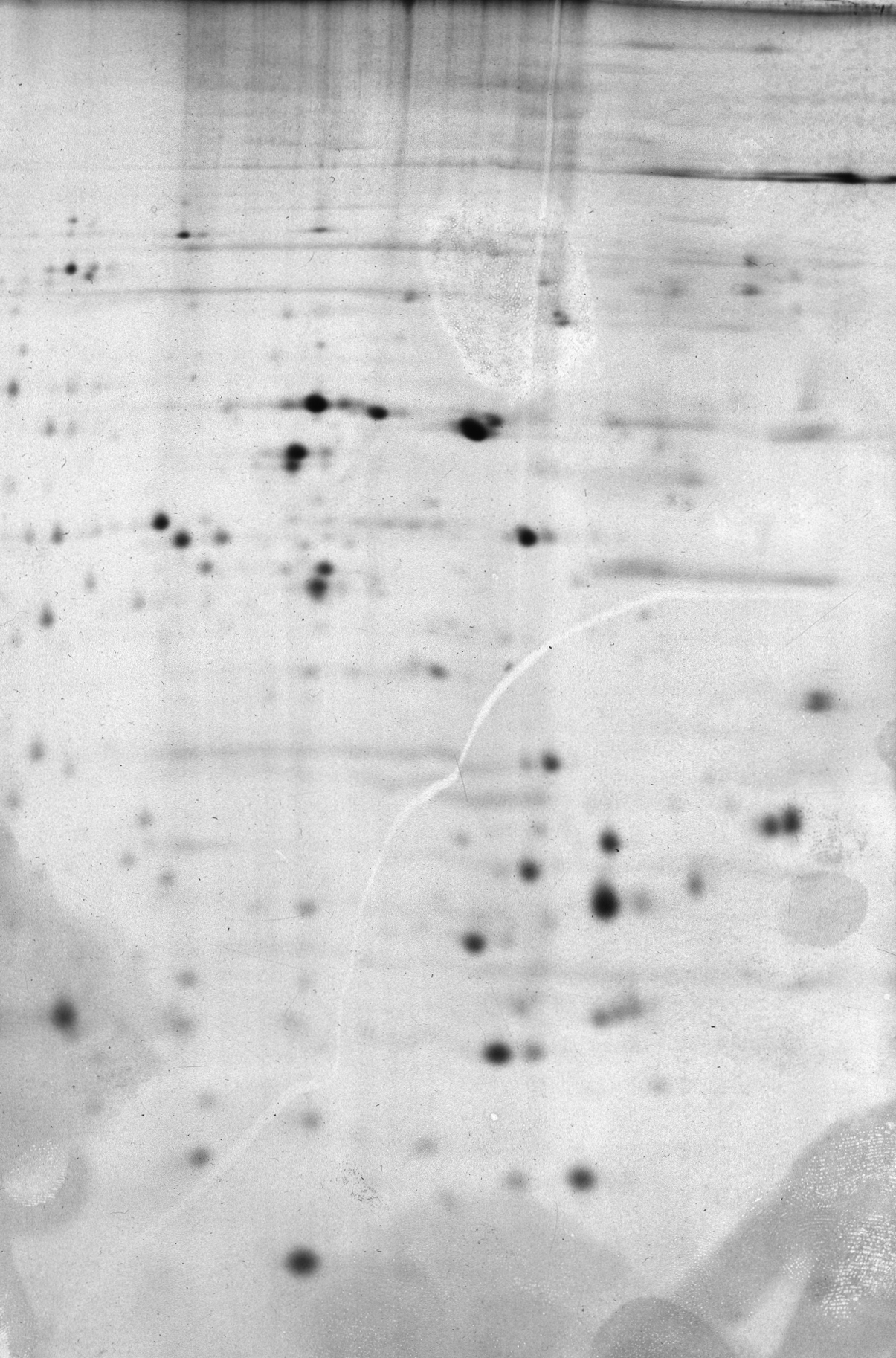

Supplement: Additional file 17 — Autoradiograph of 2D electrophoresis profile of metabolically labelled proteins from iRBC ghosts from 3D7 (see figure 6). [file 1475-2875-5-67-S17.tiff]

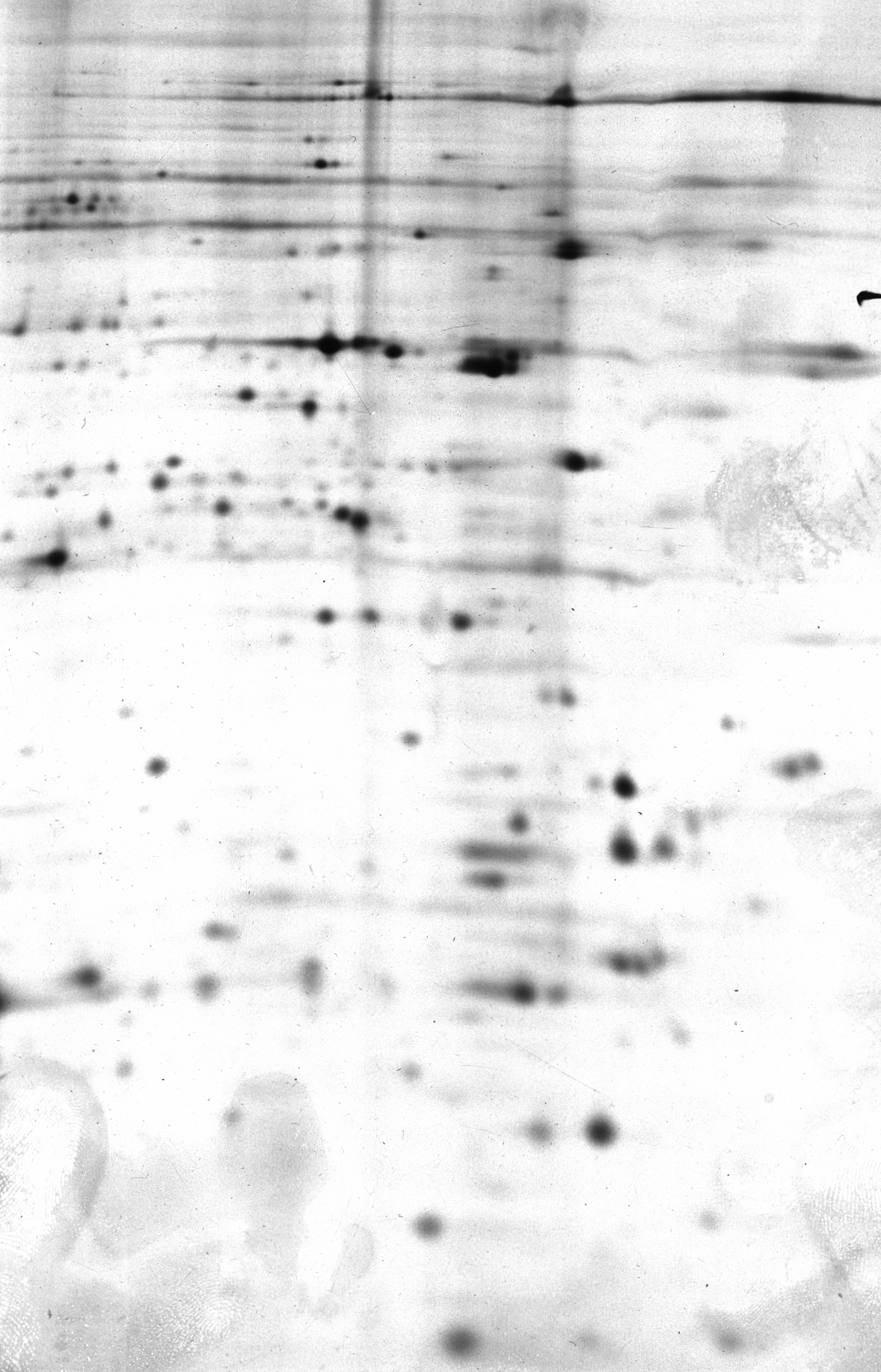

Supplement: Additional file 18 — Autoradiograph of 2D electrophoresis profile of metabolically labelled proteins from iRBC ghosts from A4 (see figure 6). [file 1475-2875-5-67-S18.tiff]

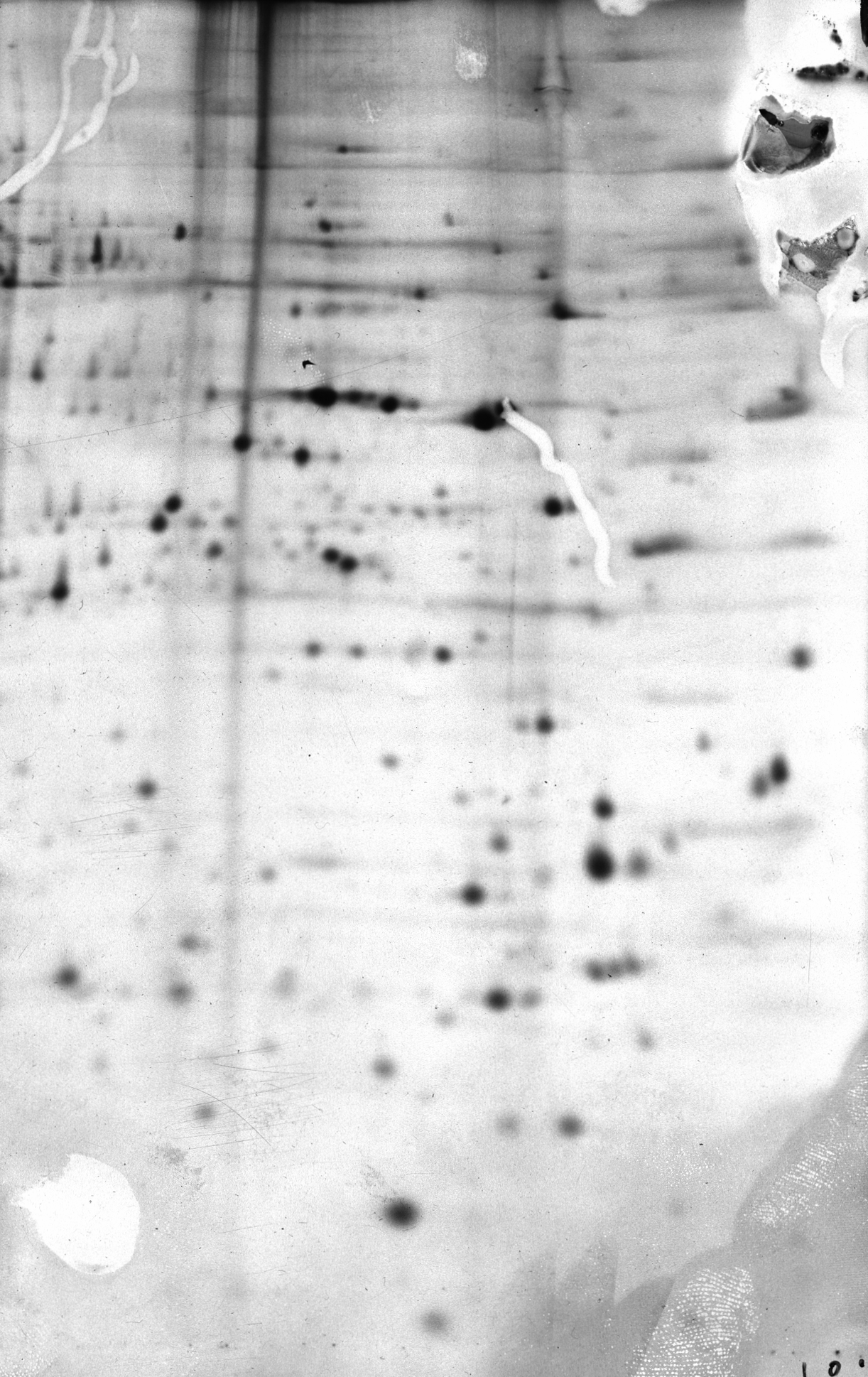

Supplement: Additional file 19 — Autoradiograph of 2D electrophoresis profile of metabolically labelled proteins from iRBC ghosts from ItG (see figure 6). [file 1475-2875-5-67-S19.tiff]

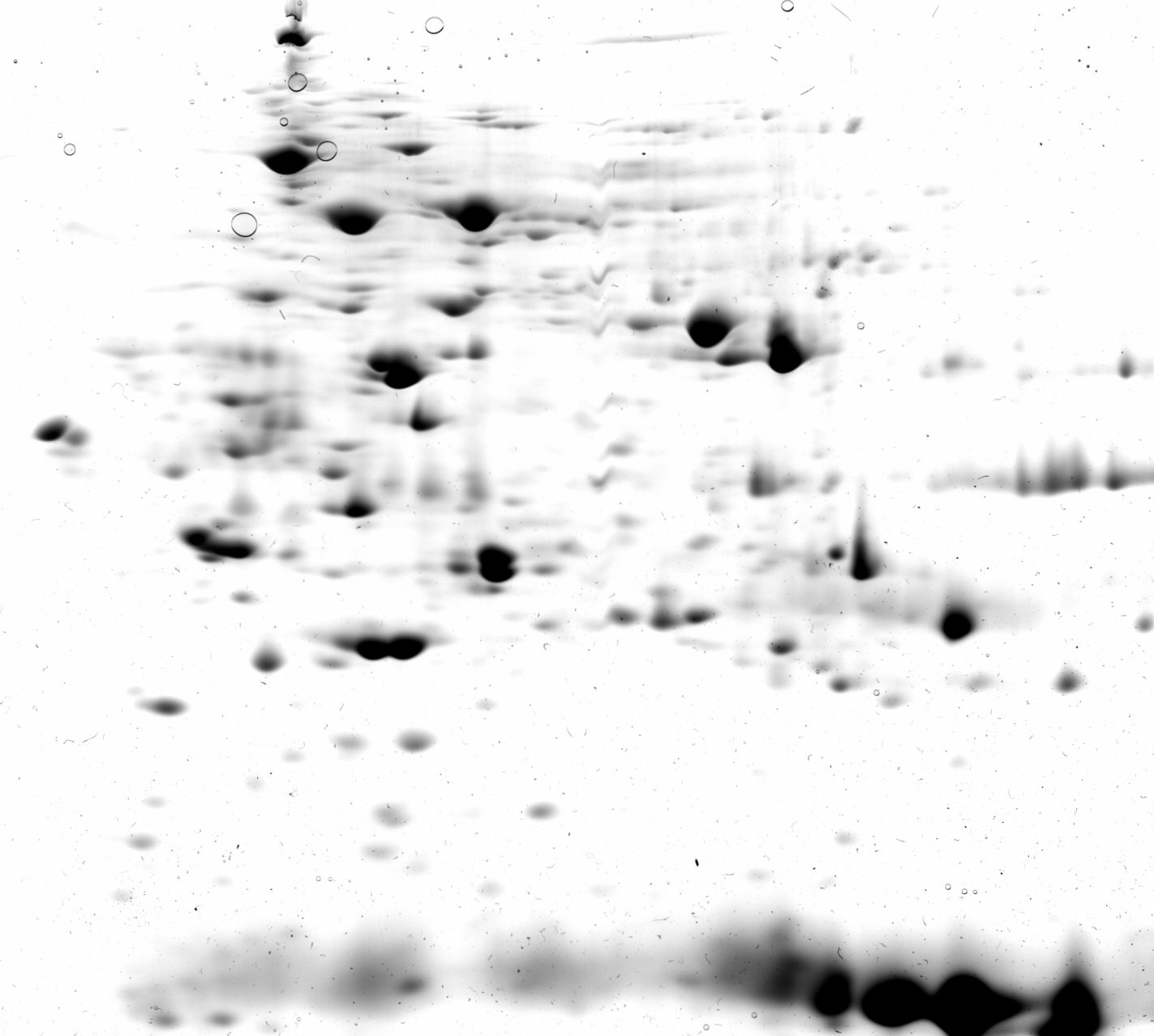

Supplement: Additional file 21 — Unannotated 2D electrophoresis profile from ItG trophozoite, free parasite fraction (see figure 8). [file 1475-2875-5-67-S21.tiff]
